# Homology-independent multiallelic disruption via CRISPR/Cas9-based knock-in yields distinct functional outcomes in human cells

**DOI:** 10.1186/s12915-018-0616-2

**Published:** 2018-12-28

**Authors:** Chenzi Zhang, Xiangjun He, Yvonne K. Kwok, Feng Wang, Junyi Xue, Hui Zhao, Kin Wah Suen, Chi Chiu Wang, Jianwei Ren, George G. Chen, Paul B. S. Lai, Jiangchao Li, Yin Xia, Andrew M. Chan, Wai-Yee Chan, Bo Feng

**Affiliations:** 1 School of Biomedical Sciences, CUHK-GIBH CAS Joint Laboratory on Stem Cell and Regenerative Medicine, The Chinese University of Hong Kong, Shatin, Hong Kong, Special Administrative Region of China; 2 Department of Obstetrics and Gynaecology, The Chinese University of Hong Kong, Shatin, Hong Kong, Special Administrative Region of China; 3 Institute for Tissue Engineering and Regenerative Medicine (iTERM), The Chinese University of Hong Kong, Shatin, Hong Kong, Special Administrative Region of China; 4 SBS Core Laboratory, CUHK Shenzhen Research Institute, Shenzhen, 518057 China; 5 Li Ka Shing Institute of Health Sciences, The Chinese University of Hong Kong, Shatin, Hong Kong, Special Administrative Region of China; 6 Department of Surgery, The Chinese University of Hong Kong, Shatin, Hong Kong, Special Administrative Region of China; 7 State Key Laboratory in Oncology in South China, Faculty of Medicine, The Chinese University of Hong Kong, Shatin, Hong Kong, Special Administrative Region of China; 8 Prince of Wales Hospital, Shatin, New Territories Hong Kong, Special Administrative Region of China; 9 Vascular Biology Research Institute, Guangdong Pharmaceutical University, Guangzhou, Guangdong 510006 People’s Republic of China; 10 Guangzhou Institute of Biomedicine and Health, Chinese Academy of Sciences, Guangzhou, 510530 China

**Keywords:** Multiallelic gene disruption, Homology-independent knock-in, Loss-of-function, Hyperploid cells, *UlK1*, *FAT10*, *CtIP*

## Abstract

**Background:**

Cultured human cells are pivotal models to study human gene functions, but introducing complete loss of function in diploid or aneuploid cells has been a challenge. The recently developed CRISPR/Cas9-mediated homology-independent knock-in approach permits targeted insertion of large DNA at high efficiency, providing a tool for insertional disruption of a selected gene. Pioneer studies have showed promising results, but the current methodology is still suboptimal and functional outcomes have not been well examined. Taking advantage of the promoterless fluorescence reporter systems established in our previous study, here, we further investigated potentials of this new insertional gene disruption approach and examined its functional outcomes.

**Results:**

Exemplified by using hyperploid LO2 cells, we demonstrated that simultaneous knock-in of dual fluorescence reporters through CRISPR/Cas9-induced homology-independent DNA repair permitted one-step generation of cells carrying complete disruption of target genes at multiple alleles. Through knocking-in at coding exons, we generated stable single-cell clones carrying complete disruption of *ULK1* gene at all four alleles, lacking intact *FAT10* in all three alleles, or devoid of intact *CtIP* at both alleles. We have confirmed the depletion of *ULK1* and *FAT10* transcripts as well as corresponding proteins in the obtained cell clones. Moreover, consistent with previous reports, we observed impaired mitophagy in *ULK1*−/− cells and attenuated cytokine-induced cell death in *FAT10*−/− clones. However, our analysis showed that single-cell clones carrying complete disruption of *CtIP* gene at both alleles preserved in-frame aberrant *CtIP* transcripts and produced proteins. Strikingly, the *CtIP*-disrupted clones raised through another two distinct targeting strategies also produced varied but in-frame aberrant *CtIP* transcripts. Sequencing analysis suggested that diverse DNA processing and alternative RNA splicing were involved in generating these in-frame aberrant *CtIP* transcripts, and some infrequent events were biasedly enriched among the *CtIP*-disrupted cell clones.

**Conclusion:**

Multiallelic gene disruption could be readily introduced through CRISPR/Cas9-induced homology-independent knock-in of dual fluorescence reporters followed by direct tracing and cell isolation. Robust cellular mechanisms exist to spare essential genes from loss-of-function modifications, by generating partially functional transcripts through diverse DNA and RNA processing mechanisms.

**Electronic supplementary material:**

The online version of this article (10.1186/s12915-018-0616-2) contains supplementary material, which is available to authorized users.

## Background

Recent breakthroughs in engineered nucleases have marked a new era for genome editing. Three main technologies, Zinc-finger nucleases (ZFNs) [1], transcription activator-like effector nucleases (TALENs) [2], and bacterial clustered regularly interspaced short palindromic repeats (CRISPR)-associated protein 9 (Cas9) system [3, 4], employ different mechanisms to recognize target DNA and introduce site-specific double-strand breaks (DSB) with high accuracy. Among these advances, the CRISPR/Cas9 system, which was originally identified to confer adaptive immunity in bacteria and archaea, has made a particularly huge impact due to its superior simplicity, low cost, and robust performance [5–7]. By forming a complex with programmable short guide RNAs (sgRNAs), Cas9 endonuclease can be directed to any pre-selected location in complex genome through complementary base pairing [3, 4]. Subsequent DNA cleavage by the sgRNA/Cas9 complex at a target site triggers intrinsic DNA repair, mainly via non-homologous end joining (NHEJ) or homology-directed repair (HDR) pathways, which allows scientists to introduce various genomic modifications in a site-specific manner [8, 9]. Currently, CRISPR/Cas9 system is widely used to augment HDR-based knock-in or knockout by inducing DNA breaks at selected target sites, and these strategies have enabled precise gene targeting in human pluripotent stem cells [10]. Meanwhile, CRISPR/Cas9-induced NHEJ is often exploited to generate loss-of-function effects in various cells and organisms, through introducing random insertions/deletions (indels) at a single target site or deleting large fragments by applying paired or multiple sgRNAs [3, 11, 12].

Human cell lines maintained under culture conditions are pivotal models for direct analysis of human gene functions. Since most cultured cells possess diploid or hyperploid genomes, meaning that a single gene is often presented as two or more copies in the genome, knockout or targeted disruption to introduce complete loss of function of a selected gene has been technically challenging in these cells. HDR-based strategies require cloning of homology arms specific to each target locus, and it remains tedious to modify multiple alleles to abolish target gene function completely. Whereas, NHEJ-mediated mutagenesis or deletions provide no means for enrichment and isolation of target cells; hence, non-selective clonal expansion and thorough screening analysis, which are often labor-intensive, are required to identify target cells harboring desired genome modifications.

Recently, studies have exploited the NHEJ repair mechanism to knock-in DNA at CRISPR-induced DSBs in both zebrafish and mammalian cells [13–15]. By targeting constitutively expressed house-keeping gene *GAPDH* at 3′-UTR using promoterless fluorescence reporters, we directly compared frequencies of knock-in mediated by CRISPR-induced NHEJ and HDR repair mechanisms [16]. We found that knock-in via CRISPR/Cas9-induced NHEJ is superior to the commonly used HDR-based method in all human cell lines examined [16]. Soon after, Zhou et al. applied this homology-independent knock-in strategy to introduce antibiotics/toxin resistance, and they successfully enriched target cells carrying desired gene disruption through drug selection [17]. However, drug selection often takes long time, and the effect varies among different cell types. Furthermore, functional outcomes from these targeted gene disruptions have not been examined [17].

In order to fully harness the recent technologies for targeted gene disruption, we took advantage of our previously established promoterless fluorescence reporter systems which produce signals only upon correct integrations, thus allowing direct tracing and cell isolation, and employed homology-independent knock-in of dual-reporters, to introduce multiallelic gene disruption in this study.

## Results

### Insertional disruption of GFP transgene via NHEJ-based knock-in

**Fig. 1:**
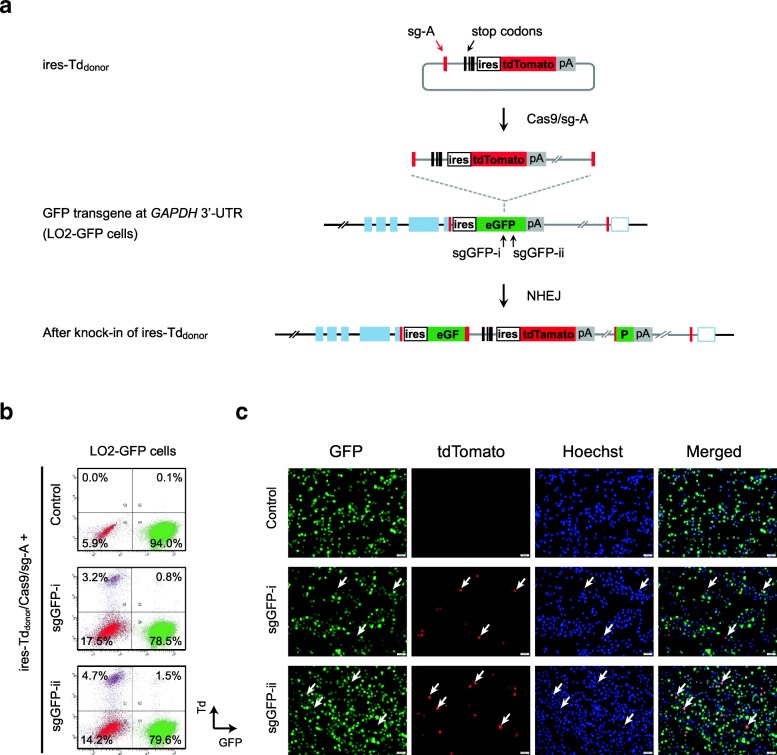
Insertional disruption of GFP transgene via NHEJ-based knock-in. **a** Schematic for NHEJ-based homology-independent knock-in of ires-Td reporter at the GFP transgene in LO2-GFP cells*.* sgGFP-i and sgGFP-ii are two different sgRNAs targeting GFP coding sequence. Shown are GFP transgene integrated at *GAPDH* locus, before and after the knock-in of ires-Td reporter. **b** FACS plots obtained after cotransfection of ires-Td_donor_/Cas9/sg-A with sgGFP-i or sgGFP-ii in LO2-GFP cells. GFP^+^ cells are gated to the right, and Td^+^ cells are gated to the top in each plot. The control without sgRNA to GFP is shown. **c** Fluorescence images showing the expression of GFP transgene as well as newly integrated tdTomato reporter. Nuclei were stained using Hoechst. Arrows indicate the cells that have acquired tdTomato expression but lost the GFP expression. Scale bars = 50 μm

To verify if NHEJ-based knock-in could introduce reporter expression and trace disruption of target gene at the same time, we performed a proof-of-principle experiment. We employed LO2-GFP cells generated previously [16] and constructed two different sgRNAs to target the constitutively expressed GFP transgene. To trace the new NHEJ knock-in events, we constructed a new donor that carry ires-tdTomato (ires-Td) together with a sg-A target site at its 5′ end, termed ires-Td_donor_ (Fig. 1a). The sg-A is a previously established sgRNA targeting non-mammalian sequence [16]. Together with Cas9, it will introduce DSB in the donor carrying corresponding target sequence for subsequent integration [16]. Indeed, after cotransfection of the ires-Td_donor_/Cas9/sg-A with either sgRNA targeting GFP, we detected a distinct Td^+^/GFP^−^ population in company with a reduction in GFP^+^ fraction, by fluorescence-activated cell sorting (FACS) (Fig. 1b). Fluorescence imaging further confirmed that the expression of GFP and tdTomato were largely exclusive to each other among the transfected cells (Fig. 1c). These results indicate that NHEJ-mediated knock-in of ires-Td reporter could be applied to enrich the disruption of GFP transgene.

### Human LO2 cells carry hyperploid genome

Unlike the GFP transgene which was present as a single copy in the above LO2-GFP cells, cultured cell lines often carry diploid or complex aneuploidy genomes, which poses additional challenges to complete disruption of a selected gene in these cells. We performed cytogenetic characterization of the wild-type LO2 cells. Chromosome number analysis revealed that LO2 cells carry hyperploid genome. Majority of the cells contained 68 (32.4%) or 66 chromosomes (23.0%, *n* = 74) (Additional file 1: Figure S1a).

Subsequently, we performed fluorescence in situ hybridization (FISH) analysis to assess the copy numbers of specific genes. Here, we selected four endogenous gene loci, *ULK1*, *FAT10*, *CtIP*, and *GAPDH* for examination*. ULK1* gene is a mammalian homologue of *Atg1*, which is known to be involved in autophagy and autophagy-mediated clearance of damaged mitochondria, a process called mitophagy [18–20]. *ULK1* is often non-essential to cell survival under normal culture conditions. *FAT10* encodes proteins mediating cellular responses to inflammation, and it is activated only upon exposure to inflammatory cytokine such as TNFα and/or INFγ [21]. The CtIP protein is known to cooperate with MRE11-RAD50-NBS1 (MRN) complex to play a pivotal role in DNA repair [22], and it is critical to cell growth as well as early development in mice [23, 24]. The *GAPDH* gene is included as control due to its universal and constitutive expression.

Our FISH analysis on these loci showed that copy numbers are varied among different genomic loci in LO2 cells. *CtIP* gene were presented at two copies, while three copies of *GAPDH* and *FAT10*, as well as four copies of *ULK1* loci, were detected (Additional file 1: Figure S1b; Table S1). Together with the FISH analyses using reference probes targeting near-centromere or sub-telomere regions in corresponding chromosomes (Additional file 1: Table S1), our results reflected complicated chromosome duplications and translocations (Additional file 1: Figure S1b). The chromosome 6 (Chr6) and chromosome 12 (Chr12) were trisomy. *FAT10* and *GAPDH* were detected within their original locations in Chr6 and Chr12, respectively, and they both were presented at three copies. Among the four *ULK1* loci detected, two remained within their original locations in Chr12, while the other two copies were detected outside Chr12 (Additional file 1: Figure S1b).

### NHEJ-based knock-in of ires-GFP at coding exons permits reporter expression

In order to trace insertional disruption of endogenous genes, we next assessed whether knock-in of ires-GFP at coding exons in *ULK1*, *FAT10*, *CtIP*, and *GAPDH* genes could result in detectable reporter expression to allow subsequent tracing.

We constructed multiple sgRNAs targeting coding exons in these genes and cotransfected ires-GFP_donor_/Cas9/sg-A with individual gene-specific sgRNAs (Additional file 1: Figure S2; left panel). Indeed, targeting *ULK1* (exon-2), *FAT10 (*exon-2), *CtIP* (exon-7), and *GAPDH* (exon-3 or exon-8), all yielded distinct fractions of GFP^+^ cells (Additional file 1: Figure S2; right panel). Moreover, consistent with previous reports showing that *FAT10* is weakly expressed in general but rapidly activated upon stimulation by TNFα and INFγ [21], GFP^+^ cells produced by targeting *FAT10 (*exon-2) could be clearly observed only in the presence of TNFα and INFγ (Additional file 1: Figure S2; right panel).

### Simultaneous knock-in of dual reporters allowed tracing and enrichment of complete disruption at *ULK1* and *FAT10* genes

**Fig. 2:**
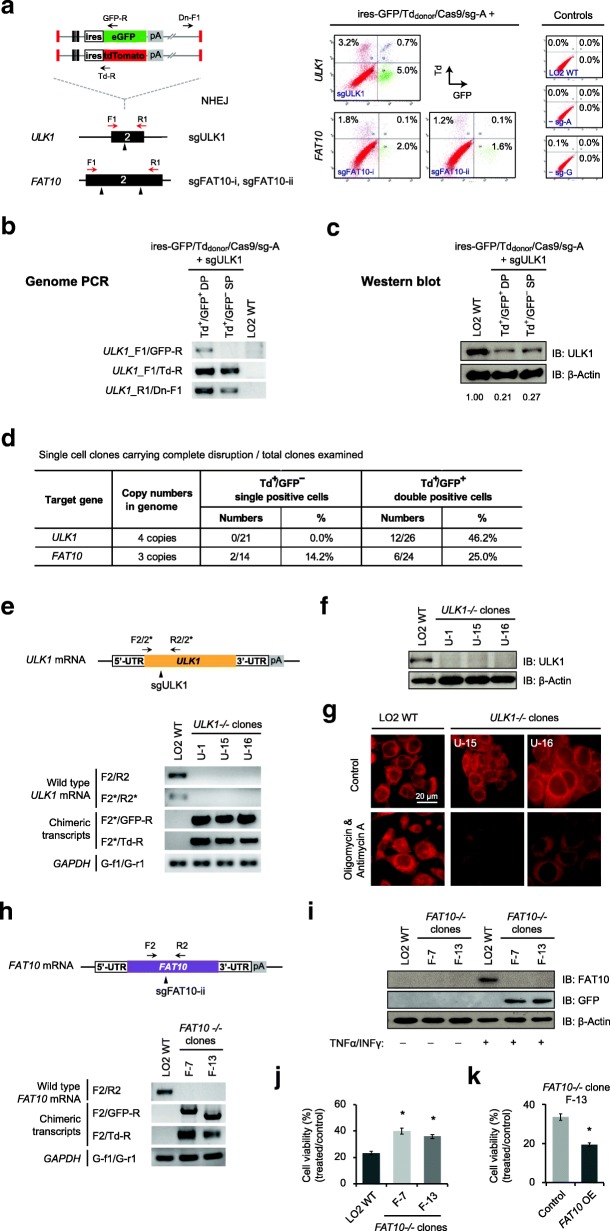
Complete disruption of *ULK1* and *FAT10* genes through simultaneous knock-in of dual reporters. **a** Schematic for dual reporter knock-in at *ULK1* and *FAT10* using ires-GFP/Td_donor_ (left), and corresponding FACS data obtained in LO2 cells (right). sgFAT10-i and sgFAT10-ii represent two different sgRNAs targeting *FAT10* exon-2. GFP^+^ cells are gated to the right, while Td^+^ cells are gated to the top. sg-G represents any sgRNA targeting *ULK1* or *FAT10* genes. Controls without sg-A or sg-G are shown. **b** Genome PCR analysis of pooled Td^+^/GFP^+^ and Td^+^/GFP^−^ cells collected from the targeting at *ULK1*. Primer binding sites are shown in **a**. **c** Western blot analysis of sorted Td+ /GFP+ and Td+ /GFP− cell populations targeted for *ULK1.* Numbers shown are ULK1 protein normalized to β-actin.  **d** Numbers of single cell clones analyzed and success rates of complete disruption from the targeting at *ULK1 or FAT10*. **e** Schematic of *ULK1* mRNA and primer binding sites (top), and corresponding RT-PCR results (bottom). *GAPDH* was included as control. **f** Western blot analysis of *ULK1*−/− clones, using antibodies against ULK1 and β-Actin. **g** Mitochondria staining with MytoTracker Red in wild-type LO2 cells and *ULK1*−/− clones. Shown are images taken after treatment with Oligomycin (10 μM) and Antimycin A (1 μM) for 24 h. Untreated cells were included as controls. Scale bars = 20 μm. **h** Schematic of *FAT10* mRNA and primer binding sites (top), and RT-PCR analysis (bottom). Cell samples were treated with TNFα and INFγ. *GAPDH* was included as control. **i** Western blot analysis of *FAT10−/−* clones, using antibodies against FAT10, GFP and β-Actin. Cells were treated with TNFα and INFγ. Untreated cells were included as control. **j** Cell viability measured using MTT assays. Shown are percentages of growth rate after treatment with TNFα and INFγ, compared to untreated cells. The measurements were done in wild-type LO2 cells and *FAT10−/−* clones. **k** Reversal of the increased cell viability in *FAT10−/−* clone F-13 after overexpression of *FAT10* cDNA. Cell viability measured using MTT assays. Each column represents the mean ± s.d. of the six replicates in **j**, or of the four replicates in **k** (Additional file 2: Individual data values for Fig. 2j and k). *p ≤ 0.05

Next, we cotransfected both ires-GFP_donor_ and ires-Td_donor_, together with Cas9/sg-A and individual gene-specific sgRNAs. Targeting *ULK1* exon-2 and *FAT10* exon-2 yielded single-positive cells expressing either reporter or distinct Td^+^/GFP^+^ double-positive cells as showed by FACS analysis (Fig. 2a). We sorted the Td^+^/GFP^+^ double-positive and Td^+^/GFP^−^ single-positive cells from the targeting at *ULK1* to verify the enrichment of gene disruption. Indeed, we detected integration of GFP and Td reporters at the pre-selected target site in *ULK1* exon-2 by genome PCR and confirmed that the reporter integrations were well correlated with the expressions (Fig. 2b). Moreover, we detected significant decrease of ULK1 proteins by western blot. Given that each reporter expression indicates integration/disruption of at least one *ULK1* allele, we detected more reduction of ULK1 protein among Td^+^/GFP^+^ double-positive cells compared to that in Td^+^/GFP^−^ single-positive cells (Fig. 2c).

We raised single-cell clones from the collected Td^+^/GFP^−^ and Td^+^/GFP^+^ cells and performed genome PCR analysis on individual clones. Strikingly, among 26 clones raised from Td^+^/GFP^+^ double-positive cells, we confirmed 12 clones to be devoid of any intact *ULK1* allele (Fig. 2d), meaning the success rate of establishing complete disruption of *ULK1* gene at all four alleles by dual-reporter tracing was around 46.2% (Fig. 2d; Additional file 1: Figure S3a). Contrarily, out of the 21 clones raised from Td^+^/GFP^−^ single-positive cells, none showed complete disruption of *ULK1* at all four alleles (Fig. 2d). Similarly, we analyzed single cell clones generated from the targeting at *FAT10* exon-2. From 24 clones raised from Td^+^/GFP^+^ double-positive cells, we identified 6 clones (25.0%) lacking intact *FAT10* alleles, whereas among 14 clones raised from Td^+^/GFP^−^ single-positive cells, we confirmed two clones with complete disruption (14.2%) (Fig. 2d; Additional file 1: Figure S3b). Collectively, these data indicate that NHEJ-based knock-in of dual fluorescence reporters could directly trace and facilitate enrichment of cells with multiallelic gene disruption.

It is worth noting that RT-PCR analysis on the three selected *ULK1*−/− clones further confirmed the absence of intact *ULK1* transcripts, as well as the presence of chimeric transcripts yielded from the integration of ires-GFP and ires-Td reporters at the target site (Fig. 2e). Meanwhile, western blot analysis verified the loss of ULK1 protein in these clones (Fig. 2f). More interestingly, we found that challenging these *ULK1*−/− cells with mitochondrial toxin Oligomycin and Antimycin A resulted in rapid mitochondria damage and depolarization, while the wild-type LO2 cells retained relative normal mitochondria as detected by MitoTracker Red (Fig. 2g). This result is consistent with the previous study on ULK1 in mitophagy, indicating that ULK1 plays an important role in clearing damaged mitochondria through autophagy and maintaining mitochondrial homeostasis [18, 20].

Similarly, we confirmed the depletion of intact endogenous *FAT10* mRNA as well as the acquisition of chimeric transcripts in two selected *FAT10*−/− clones (Fig. 2h). Western blot analysis further verified that these *FAT10*−/− clones failed to produce FAT10 proteins upon induction by TNFα and INFγ; instead, they produced GFP proteins from the integrated transgene (Fig. 2i). Consistent with previous reports that activation of *FAT10* caused cell apoptosis [25], we found that the *FAT10*−/− cells showed increased cell survival compared to wild-type LO2 cells upon the treatment with TNFα and INFγ (Fig. 2j). Moreover, the increased cell survival in the *FAT10*−/− cells could be abolished by ectopic expression of *FAT10*, indicating that FAT10 was indeed involved in the TNFα/INFγ-induced cell death (Fig. 2k). Altogether, these results supported that NHEJ-based simultaneous knock-in of dual fluorescence reporters successfully enriched and established targeted disruptions of *ULK1* and *FAT10* genes at high efficiency.

### Complete disruption of *CtIP* gene resulted in cells carrying in-frame aberrant transcripts

**Fig. 3:**
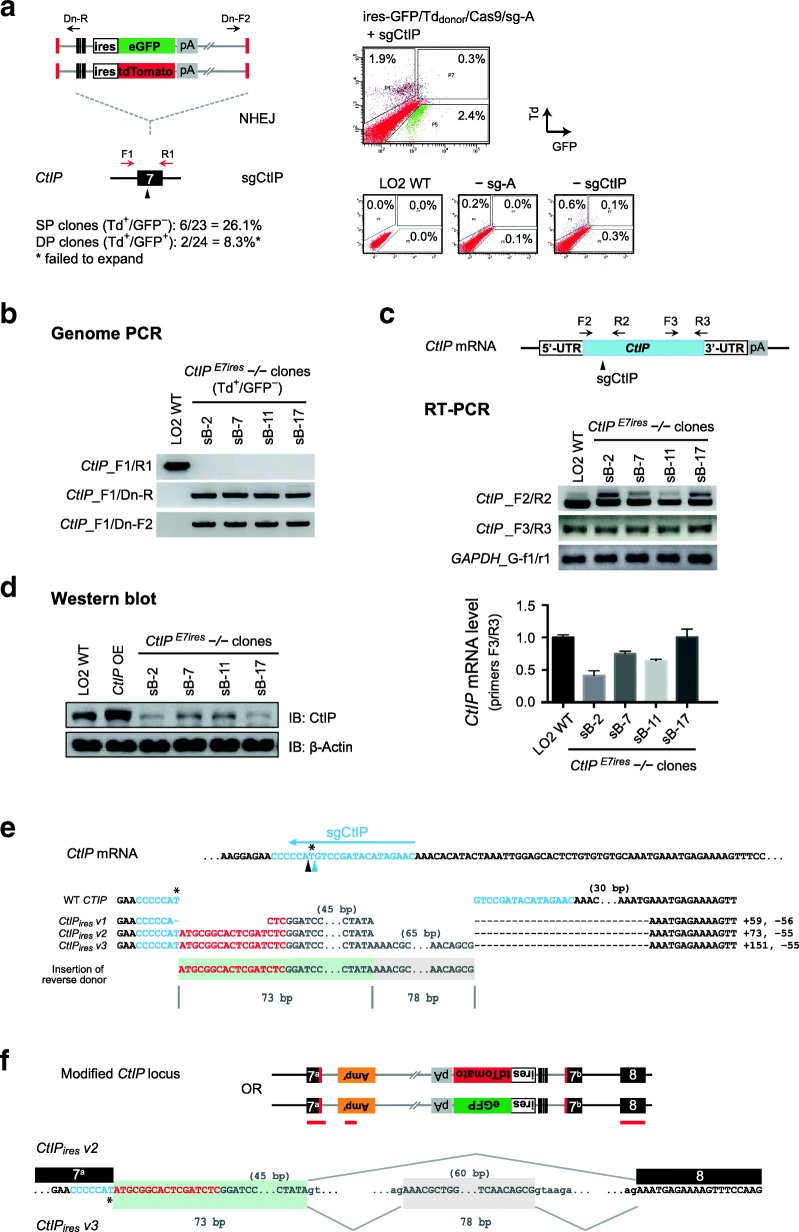
Targeted disruption at *CtIP* exon-7 yielded cells carrying in-frame variant transcripts. **a** Schematic for dual reporter knock-in at *CtIP* using ires-GFP/Td_donor_ (left), and corresponding FACS data obtained in LO2 cells (right). GFP^+^ cells are gated to the right, while Td^+^ cells are gated to the top. Controls without sg-A or sgCtIP are included in right panel. Numbers of single cell clones analyzed and success rates of complete disruption at *CtIP* are shown. **b** Genome PCR analysis of *CtIP*^*E7ires*^−/− clones raised from the Td^+^/GFP^−^ single-positive (SP) cells**.** Primer binding sites are shown in **a**. **c** Schematic of *CtIP* mRNA, sgCtIP target site, and primer binding positions (top); and gel electrophoresis of RT-PCR from selected *CtIP*^*E7ires*^−/− clones (middle), and quantitative analysis by real-time RT-PCR using primers *CtIP*_F3/R3 (bottom) (Additional file 2: Individual data values for Fig. 3c). **d** Western blot analysis of selected *CtIP*^*E7ires*^−/− clones. Cells transiently transfected with *CtIP* cDNA were included. OE:overexpression. **e** Junction sequences of three aberrant *CtIP*_*ires*_ transcripts amplified using primers *CtIP*_F2/R2 in **c**. The sg-A target sequence from donor is shown in red, and sgCtIP target sequence from genome is in blue. The cleavage sites at the 3rd and 4th nucleotide upstream of PAM in sgCtIP target sequence are indicated with black and blue arrowheads respectively. Other donor sequences are in grey, while other sequences from *CtIP* genome locus are in black. Two short fragments originated from donor vectors are highlighted in light green and beige. The numbers of base pairs omitted are indicated in brackets. **f** Schematic showing the modified *CtIP* alleles harboring reversely integrated ires-Td_donor_ or ires-GFP_donor_ (top). Red bars below indicated the positions of sequences detected in the aberrant *CtIP*_*ires*_ transcripts. Sequences showing cryptic splice sites and the splicing events involved in producing the aberrant *CtIP*_*ires*_ transcripts (bottom)

Interestingly, while the NHEJ-based simultaneous knock-in of dual reporters successfully established stable clones carrying complete disruption of *ULK1* at four alleles or devoid of *FAT10* at three alleles in the hyperploid genome, targeted disruption at the biallelic *CtIP* gene yielded complicated results. Cotransfection of both ires-GFP_donor_ and ires-Td_donor_, together with Cas9/sg-A and sgCtIP targeting *CtIP* exon-7, produced detectable GFP and tdTomato signals to support cell sorting (Fig. 3a). However, among 24 single-cell clones raised from Td^+^/GFP^+^ double-positive cells, only two clones were found to carry *CtIP*-disruption and they failed to expand subsequently (8.3%). Rather surprisingly, we identified 6 *CtIP*-disrupted clones out of 23 clones raised from Td^+^/GFP^−^ single-positive cells, indicating a success rate higher than that from the double-positive cells, at 26.1% (Additional file 1: Figure S4a).

We selectively analyzed four clones isolated from the Td^+^/GFP^−^ cells, termed *CtIP*^*E*7ires^−/− clones, and detected targeted insertions of donors at both forward and reverse orientations in each clone (Fig. 3b). Surprisingly, despite of the absence of intact *CtIP* alleles, significant amounts of *CtIP* transcripts were detected in these cells, using primers matching to *CtIP* mRNA at both the target and non-target regions (Fig. 3c). Moreover, reduced yet substantial amounts of CtIP proteins were detected in all four clones by western blot (Fig. 3d).

More interestingly, sequencing analysis revealed three different aberrant transcripts (*CtIP*_*ires*_ *v1–3)* from these clones, and they all preserved the original reading frame of *CtIP* mRNA (Fig. 3e). Sequence alignment suggested that all these *CtIP*_*ires*_ transcripts were produced from reversely integrated donors, but not from the forward integrations which were confirmed by genome PCR and produced Td^+^ signal detected by FACS analysis (Fig. 3a, b). One cryptic splice donor (SD) site from reverse donor backbone likely paired with the splice acceptor (SA) site of *CtIP* exon-8, resulting in a short insertion of the donor sequences (Fig. 3e, f; shade in light green) together with the deletion of 3′-part of *CtIP* exon-7 (exon-7^b^; − 55 bp) in all three aberrant transcripts. Additionally, *CtIP*_*ires*_ *v1* carried a 15-bp deletion near the sgCtIP target site, while *CtIP*_*ires*_ *v3* contained an extra insertion (+ 78 bp), which was likely originated from the reverse donor backbone and produced through a weak splicing event (Fig. 3e, f; shades in beige). Besides the varied alternative RNA splicing, the sequencing data also indicated that *CtIP*_*ires*_ *v2* and *V3* carried sgCtIP target sequences cleaved at the 4th nucleotide instead of the commonly used 3rd nucleotide upstream of the protospacer adjacent motif (PAM) [26] (Fig. 3e, f; asterisks). The apparently biased enrichment of reverse integration and alternative DNA cleavage by Cas9/sgCtIP in all these cell clones suggested that the in-frame aberrant *CtIP*_*ires*_ transcripts were likely to be functional and beneficial to cell survival. This is consistent with the reported CtIP function in DNA repair, and it explains why the double-positive *CtIP*-disrupted clones failed to expand eventually.

### Disrupting *CtIP* gene using distinct donors yielded cells with different in-frame transcripts

**Fig. 4:**
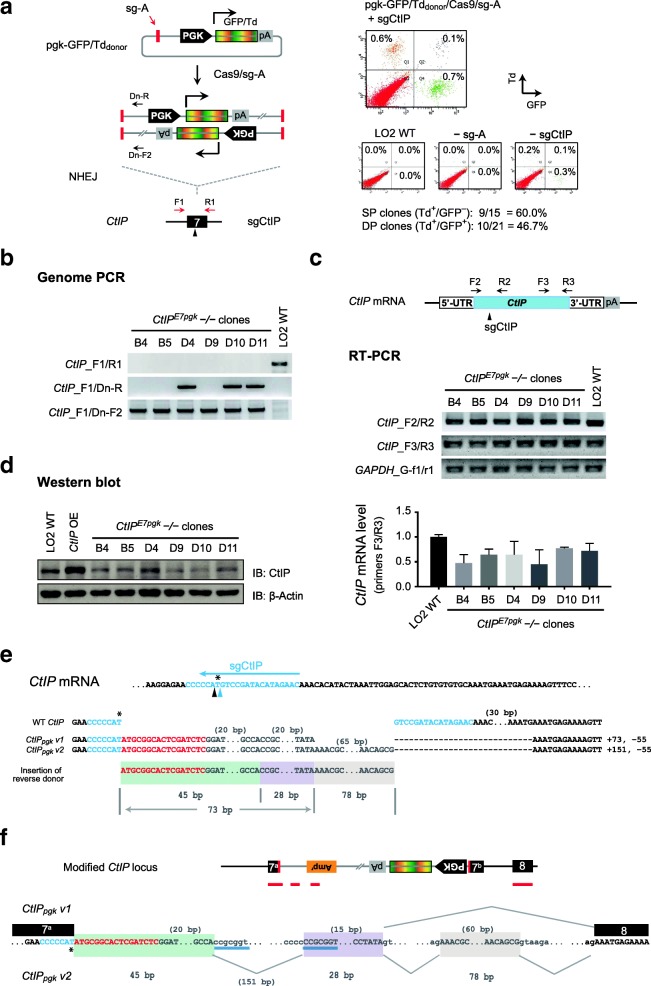
Targeted knock-in of pgk-GFP/Td reporters at *CtIP* exon-7 yielded cells carrying distinct in-frame transcripts. **a** Schematic for dual reporter knock-in at *CtIP* using pgk-GFP/Td_donor_ (left), and corresponding FACS data obtained in LO2 cells (right). GFP^+^ cells are gated to the right, while Td^+^ cells are gated to the top. Controls without sg-A or sgCtIP are included. Numbers of single-cell clones analyzed and success rates of complete disruption are shown. **b** Genome PCR showing *CtIP* disruption, forward and reserve integrations of donors, in selected *CtIP*^*E7pgk*^ −/− clones raised in **a**. **c** RT-PCR analysis of the selected *CtIP*^*E7pgk*^ −/− clones. Shown are schematic of *CtIP* mRNA, sgCtIP target site, and primer binding positions (top), gel electrophoresis of RT-PCR products (middle), and quantitative RT-PCR analysis using primers *CtIP*_F3/R3 (bottom) (Additional file 2: Individual data values for Fig. 4c). **d** Western blot analysis of the selected *CtIP*^*E7pgk*^ −/− clones using antibodies against CtIP and β-actin. Cells transiently transfected with *CtIP* cDNA were included as positive control. OE, overexpression. **e** Junction sequences of two aberrant *CtIP*_*pgk*_ transcripts amplified using primers *CtIP*_F2/R2 in **c**. The sg-A target sequence is in red, and sgCtIP target sequence from genome is in blue. The cleavage sites at the 3rd and 4th nucleotide upstream of PAM in sgCtIP target sequence are indicated with black and blue arrowheads respectively. Other donor sequences are in grey, while other sequences from *CtIP* genome locus are in black. Three short fragments originated from donors are highlighted with shades in different colors. The numbers of base pairs omitted are indicated in brackets. **f** Schematic for the modified *CtIP* allele harboring reversely integrated pgk-GFP_donor_ or pgk-Td_donor_ (top). Red bars below indicated the positions of sequences detected in the aberrant *CtIP*_*pgk*_ transcripts. Sequences showing cryptic splice sites and the splicing events involved in producing the aberrant *CtIP*_*pgk*_ transcripts detected (bottom)

To examine the generality of above observations, we employed distinct donors pgk-GFP/Td_donor_ to target *CtIP* exon-7 for gene disruption (Fig. 4a, left panel). Differently from the promoterless donors used above, pgk-GFP/Td_donor_ carried an active PGK promoter and supported constitutive reporter expression regardless the orientation of insertion [16]. Similarly, we performed cotransfection of dual pgk-GFP/Td_donor_ and Cas9/sg-A/sgCtIP and sorted Td^+^/GFP^−^ and Td^+^/GFP^+^ cells for clonal analysis (Fig. 4a; right panel). Among the 21 clones raised from Td^+^/GFP^+^ double-positive cells and 15 clones from Td^+^/GFP^−^ single-positive cells, we identified 10 (47.6%) and 9 (60.0%) clones respectively, to be devoid of intact *CtIP* alleles (Fig. 4a; Additional file 1: Figure S5a).

Interestingly, selective analysis of six clones raised from either double-positive cells or single-positive cells revealed that they all harbored reverse integrations (Fig. 4b), while in contrast, only three clones carried forward insertions (Fig. 4b). Similar to the targeting with ires-GFP/Td_donor_, RT-PCR analysis of these *CtIP*^*E7pgk*^ −/− clones detected substantial amounts of *CtIP* transcripts covering either the target or non-target regions (Fig. 4c), and western blot detected reduced yet substantial amounts of CtIP proteins (Fig. 4d).

Sequencing analysis showed that, again, the aberrant transcripts (*CtIP*_*pgk*_ *v1* and *v2)* were all in-frame and produced from the modified alleles carrying reverse integration (Fig. 4e, f; Additional file 1: Figure S5b). Splicing likely happened between a distinct SD site embedded in the new donor backbone and the SA site of *CtIP* exon-8 (Fig. 4f), thus resulting in an insertion of distinct donor sequences and partial deletion of *CtIP* exon-7 (exon-7^b^; − 55 bp) (Fig. 4f). Interestingly, an additional deletion (− 151 bp) was observed within the donor sequences embedded in the *CtIP*_*pgk*_ transcripts, which yielded a final insertion at 73 bp and net insertion of 18 bp, same to that in *CtIP*_*ires*_ transcripts (Fig. 4f). Verification on the vector, modified genome and aberrant transcript sequences suggested that the additional 151-bp deletion was likely to be introduced by a microhomology-based recombination event at genome level (Additional file 1: Figure S5b, c). Furthermore, the longer variant *CtIP*_*pgk*_ *v2* was found to carry an additional short insertion (+ 78 bp) from vector backbone, which was identical to that in *CtIP*_*ires*_ *v3* and likely to be produced from a similar weak splicing event (Fig. 4f; Additional file 1: Figure S5b, shade in beige). Finally, these aberrant *CtIP*_*pgk*_ transcripts also carried sgCtIP target sequences cleaved at the 4th nucleotide. Altogether, these analyses detected various combinations of DNA processing and RNA splicing, which yielded two distinct *CtIP*_*pgk*_ transcripts and both were in-frame (Fig. 4e, f; Additional file 1: Figure S5b, c). These data are consistent with the results obtained above using ires-GFP/Td_donor_, suggesting that the escapee cells might be generated under survival stress through producing various partially functional *CtIP* transcripts and proteins.

### Targeting *CtIP* 5′-UTR also produced cells escaping from complete protein depletion

To prove the generality of above observations, we also targeted proximal 5′-UTR of *CtIP* to introduce gene disruption via the NHEJ-based knock-in approach. We generated new donors by deleting the ires element from previous constructs and termed them 5′GFP/Td_donor_. Cotransfection of the 5′GFP_donor_/Cas9/sg-A together with individual sgRNAs targeting 5′-UTR of *GAPDH* or *CtIP* genes confirmed that targeting at two out of the three selected target sites yielded significant fractions of GFP-positive cells (Additional file 1: Figure S6a).

**Fig. 5:**
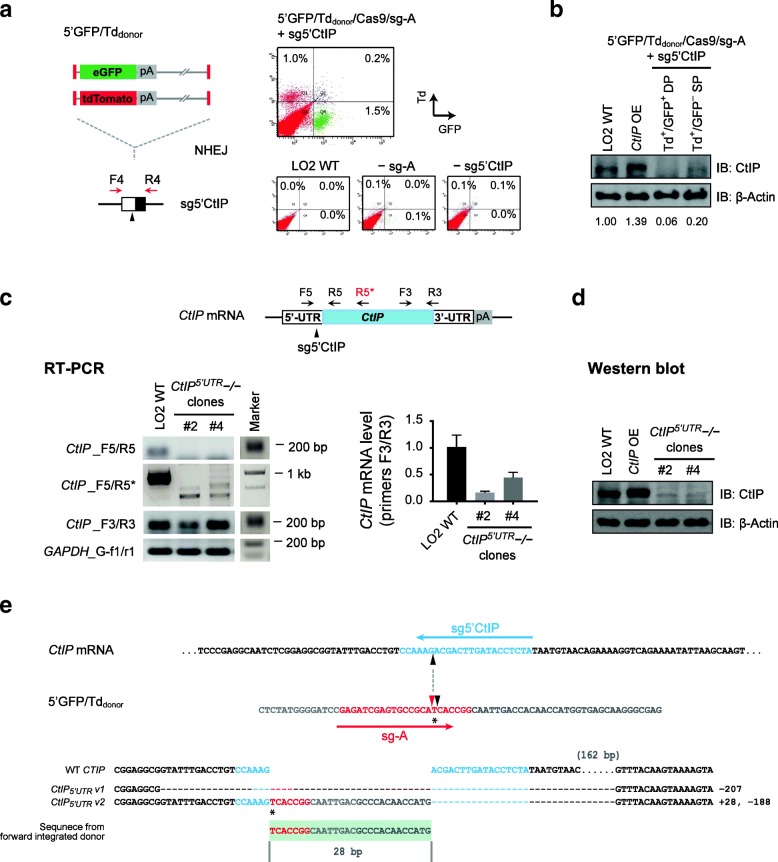
Targeted disruption at *CtIP* 5′-UTR also produced cells carrying splice variant transcripts. **a** Schematic for simultaneous knock-in of 5’GFP_donor_ and 5′Td_donor_ at *CtIP* 5′-UTR (left); and FACS plot obtained in LO2 cells (right). GFP^+^ cells are gated to the right, while Td^+^ cells are gated to the top. Controls without sg-A or sg5′CtIP are shown. **b** Western blot analysis of pooled Td^+^/GFP^+^ double-positive (DP) and Td^+^/GFP^−^ single-positive (SP) cells collected from **a**. Cells transiently transfected with *CtIP* cDNA were included. Numbers shown are CtIP protein levels normalized to β-actin. OE, overexpression. **c** RT-PCR analysis of selected *CtIP*^*5’UTR*^ −/− clones. Shown are schematic of *CtIP* mRNA, sg5′CtIP target site, and primer binding positions (top), gel electrophoresis of RT-PCR products (bottom left), and quantitative RT-PCR analysis using primers *CtIP*_F3/R3 (bottom right) (Additional file 2: Individual data values for Fig. 5c). **d** Western blot analysis of the selected *CtIP*^*5*′*UTR*^ −/− clones. Cells transiently transfected with *CtIP* cDNA were included as positive control. OE, overexpression. **e** Sequences of the RT-PCR products amplified from aberrant *CtIP*_*5*′*UTR*_ transcripts with primers *CtIP*_F5/R5* in **c**. Shown are junction sequences of two aberrant *CtIP*_*5*′*UTR*_ transcripts. The sg-A target sequence is in red, and sg5′CtIP target sequence at *CtIP* 5′-UTR is in blue. The cleavage sites at the 3rd and 4th nucleotide at upstream of PAM in sg-A target sequence are indicated with black and red arrowheads respectively. Other donor sequences are in grey, while other sequences from *CtIP* genome locus are in black. The short fragment originated from donors is highlighted with shades in light green. The number of base pairs omitted is indicated in brackets

Next, we cotransfected both 5′GFP_donor_ and 5′Td_donor_ together with Cas9/sg-A /sg5′CtIP for simultaneous knock-in at *CtIP* 5′-UTR and detected a small but distinct fraction of Td^+^/GFP^+^ double-positive cells (Fig. 5a). Western blot analysis of the sorted cells confirmed significant reduction of CtIP proteins and revealed more severe reduction in the Td^+^/GFP^+^ double-positive than that in Td^+^/GFP^−^ single-positive cells (Fig. 5b).

We examined 10 single clones raised from the double-positive cells and identified 3 clones (30.0%) lacking intact *CtIP* genes (Additional file 1: Figure S6b), termed *CtIP*^*5’UTR*^−/− clones. RT-PCR confirmed the absence of intact wild-type *CtIP* transcripts in these cells (Fig. 5c; *CtIP*_F5/R5). However, using primers covering a longer region around the target site, we detected shortened aberrant transcripts (Fig. 5c; *CtIP*_F5/R5*). Consistently, substantial amount of transcripts was detected using primers to the non-target region in *CtIP* mRNA (Fig. 5c; *CtIP*_F3/R3), and western blot analysis confirmed the presence of trace CtIP proteins (Fig. 5d).

Sequencing analysis of the aberrant *CtIP*_*5’UTR*_ transcripts showed that they were produced from forwardly integrated donors (Fig. 5e; Additional file 1: Figure S6c). The integrated donor sequences were largely spliced out together with the 3′-part of *CtIP* exon-1 (exon-1^b^; 188 bp) carrying the start codon, through two cryptic splicing events. *CtIP*_*5*′*UTR*_ *v1* was likely to be produced through pairing between an endogenous cryptic SD site upstream of the sg5′CtIP target site and the SA site of *CtIP* exon-2. Hence, it lacked a large fragment (− 207 bp), including 19 bp upstream of the sg5′CtIP cleavage site and the 3′-part of exon-1 (exon-1^b^; 188 bp). *CtIP*_*5’UTR*_ v2 was probably yielded from pairing between a cryptic SD site in the forward donor backbone and the SA site of *CtIP* exon-2; thus, it carried a small insertion (+ 28 bp) of donor sequences together with the deletion of 3′-part of exon-1 (− 188 bp) (Fig. 5e; Additional file 1: Figure S6c, d). The lack of authentic start codon in these *CtIP*_*5*′*UTR*_ transcripts might lead to usage of downstream cryptic initiation codons and significantly reduced protein translation.

Collectively, with three different targeting strategies, we detected various aberrant *CtIP* transcripts produced from differently modified *CtIP* gene loci, which were all in-frame and produced altered CtIP proteins. These results are consistent with the critical function of CtIP in DNA repair, suggesting that these *CtIP*-disrupted cell clones were likely to be raised from escapee cells, which stood out from clonal expansion because of the partially functional transcripts and CtIP proteins.

## Discussion

In this study, we readily achieved multiallelic gene disruption in hyperploid human cells through insertion of dual fluorescence reporters via CRISPR/Cas9-mediated homology-independent knock-in approach and observed diverse outcomes by targeting *ULK1*, *FAT10*, and *CtIP* loci, which represented non-essential, inducible, and essential genes respectively. First, through simultaneous knock-in of GFP and tdTomato followed by FACS-based enrichment, we successfully generated single-cell clones carrying complete disruption of *ULK1* at all four alleles, lacking intact *FAT10* in all three alleles, or devoid of intact *CtIP* at both alleles in LO2 cells. In particular, the efficiency for complete disruption of all four *ULK1* alleles reached up to 46.2%. Subsequently, RT-PCR, sequencing, and western blot analyses confirmed the depletion of *ULK1* and *FAT10* transcripts as well as corresponding proteins, in the single-cell clones carrying corresponding gene disruptions. Importantly, we confirmed that the *ULK1−/−* cells generated through this approach displayed significant defect in clearing damaged mitochondria, which is consistent with the ULK1 function in mitophagy, while the *FAT10−/−* cells showed increased cell survival upon the treatment of TNFα and INFγ, which correlates well with FAT10 function in mediating apoptosis triggered by these cytokines.

Interestingly, despite the complete disruption of *CtIP* gene at both alleles was successfully achieved through three different targeting strategies, the single cell clones obtained always preserved in-frame transcript variants. Sequencing analysis revealed various aberrant *CtIP* transcripts, suggesting that they were produced through diverse DNA processing and RNA splicing processes, including alternative DNA cleavage by CRISPR/Cas9 at the 4th nucleotide upstream of PAM, microhomology-based genome DNA recombination, and different alternative RNA splicing. These results are in line with the recent findings by Mou et al. and Kapahnke et al., which detected various exon-skipping/exon-deletion events upon introduction of loss-of-function mutations using CRISPR technology [27, 28]. Towards demonstrating the generality of this phenomenon, our data provided new evidence through distinct targeting approaches and showed that a broader range of cellular mechanisms could readily participate in dynamic DNA and RNA processing to yield escapee cells under survival stress.

Collectively, our study demonstrated that the homology-independent dual-reporter knock-in approach provides a promising strategy to trace and enrich multiallelic gene disruption, which is particularly valuable to research on gene functions using diploid and hyperploid cell models. Meanwhile, together with previous studies, our data suggested that aberrant transcripts might be generated through various cellular mechanisms when disrupting genes essential to cell growth or survival, regardless of the methodology used.

Recent technological advances have significantly empowered fast-forward genetics. Massively parallel sequencing has enabled efficient discovery of unknown genetic variants associated with human diseases [29]. High-throughput genome-wide RNA interference (RNAi) screen has permitted rapid identification of causal genes responsible for specific cellular functions and phenotypes [30]. New approaches such as introduction of random indels by CRISPR/Cas9-induced NHEJ repair [11, 31, 32] or CRISPR interference (CRISPRi) mediated by fusing inactivated Cas9 to repressive KRAB domain [33, 34] have also achieved great success in unraveling genes involved in various cellular processes. Contrarily, function analysis of candidate genes, e.g., reverse genetics, has been hindered by various technical hurdles and often leads to inaccurate annotation or even misinterpretation of gene functions. The current challenges include but are not limited to incomplete depletion of target mRNA/proteins by RNAi or CRISPRi approaches, low efficiency associated with HDR-based knockout, and lack of means to trace and enrich target cells when indels or deletions are introduced via CRISPR/Cas9-coupled NHEJ repair.

In this context, the homology-independent knock-in system established by our recent study provides an efficient tool to introduce complete gene disruption through the insertion of trackable reporters [16]. The homology-free donor and non-mammalian sg-A target sequence have made this system easily applicable to any selected locus in mammalian genome [16]. Moreover, the high efficiency of NHEJ-based knock-in could permit simultaneous knock-in at multiple alleles, while usage of dual fluorescence reporters followed by FACS sorting grants direct tracing of cells carrying multiple knock-in and likely multiallelic gene disruption. In this study, we employed this approach and achieved one-step generation of cell clones devoid of intact *ULK1*, *FAT10*, or *CtIP* genes, despite of their multiple copies in the hyperploid LO2 cells. This has allowed us to quickly assess the functions of disrupted genes, as well as the efficacy and limitation of the gene-disruption strategy in the current study.

Intriguingly, while we fully confirmed the depletion of *ULK1* and *FAT10* transcripts and proteins, as well as the loss-of-function phenotypes in the single-cell clones carrying complete gene disruptions, our study detected various chimeric *CtIP* transcripts and demonstrated an apparently biased enrichment of minor events through cell survival. First, upon targeting *CtIP* exon-7 with two different types of donors, reverse integrations, which should not be enriched by FACS sorting, were confirmed in all *CtIP*^*E7ires*^−/− and *CtIP*^*E7pgk*^−/− clones examined (Fig. 3b; Fig. 4b). Moreover, despite the presence of forward integrations confirmed by FACS and genome PCR, aberrant *CtIP*_*ires*_ and *CtIP*_*pgk*_ transcripts were only recovered from the alleles carrying reverse integration in these cells (Fig. 3f; Fig. 4f). Second, we observed alternative DNA cleavage by Cas9 at the 4th nucleotide upstream of PAM, in all *CtIP*-disrupted clones generated through three different strategies (Fig. 3e, Fig. 4e and Fig. 5e). This is apparently biased because DNA cleavage at the 3rd nucleotide is known to be dominant and prevalent [3, 16]. Finally, all aberrant *CtIP* transcripts preserved the original reading frame of *CtIP* mRNA and produced detectable CtIP proteins, despite the fact that they carried integrations of different donors and were produced through different DNA processing and alternative RNA splicing (Fig. 3f; Fig. 4f; Additional file 1: Figure S6d). Given the large numbers of cryptic splice sites within the donor sequences in either orientation, as predicted by Human Splicing Finder [35], the detection of only in-frame aberrant transcripts in all *CtIP*-disrupted clones strongly suggested that they were functional. It also suggested that preserving CtIP function might be associated with a survival advantage in LO2 cells. This is consistent with the critical function of CtIP in DNA repair [36], and it is supported by previous reports showing that ablation of *CtIP* gene impaired cell proliferation and early development in mice [23, 24].

Collectively, these results are consistent with the unexpected exon-skipping/exon-deletion observed by Mou et al. and Kapahnke et al [27, 28, 37]. Meanwhile, they provide new evidence through a distinct targeting approach. Our data showing the biased enrichment of minor events with three different targeting strategies further support the generality of this phenomenon, suggesting that essential genes may be spared from loss-of-function mutation/deletion/disruption through various cellular mechanisms. Hence, more caution is needed to interpret functions of genes involved in cell growth or survival, especially using a loss-of-function approach. Altogether, these data put forward new evidence on the tremendous flexibility provided through diverse cellular mechanisms, which is likely to be critical to ensure a functional output from the numerous DNA and RNA processing events in cells.

The off-target effect of CRISPR technology has been intensively investigated and debated. Several studies provide evidence that off-target insertions or deletions were induced at low frequencies near the detection limit of deep sequencing, which likely had minimal impact on whole-genome mutation load [38–41]. Only one recent study showed unexpected high mutation rate from their in vivo work and evoked vigorous debate, but it has just been retracted by the publication group [42]. Our previous study has also examined the off-target effect of homology-independent knock-in approach [16]. In the current study, potential false-positive signals introduced by off-target integrations have been examined by the control experiments performed in the absence of sg-A or any gene-specific sgRNA targeting *ULK1*, *FAT10*, *CtIP*, or *GAPDH* (Fig. 2a, Fig. 3a, Fig. 4a and Fig. 5a; Additional file 1: Figure S2 and Figure S6a). Consistent with our previous study, compared to reporter signals produced by targeted integrations, non-specific or off-target integrations yielded only low signals negligible to the current conclusion.

## Conclusion

Multiallelic gene disruption could be readily introduced through CRISPR/Cas9-induced homology-independent knock-in of dual fluorescence reporters followed by direct tracing and cell isolation. Meanwhile, diverse DNA repair and RNA splicing mechanisms in cells may form a robust cellular mechanism to spare essential genes from loss-of-function modifications by generating partially functional transcripts. Hence, distinct functional outcomes might be yielded when targeting different genes, regardless of the strategies and tools used.

## Methods

### Cas9 and sgRNA constructs

The human codon-optimized Cas9 was a gift from George Church (Addgene # 41815) [4]. All sgRNAs used in this study were designed and constructed as previously described [4, 16, 43], and their target sequences are listed in Additional file 1: Table S2.

### Donor constructs

1. ires-GFP/Td donors: Single-cut non-homology (NH)-donor carrying a sg-A target site followed by ires-eGFP cassette was constructed in our previous study [16]. A pair of oligos carrying multiple stop codons in three different reading frames was synthesized, anealed and inserted between the sg-A target sequence and ires-eGFP in single-cut NH donor to construct the ires-GFP_donor_. The eGFP coding sequence in ires-GFP_donor_ was then replaced with the tdTomato (Td) gene to generate the ires-Td_donor_.
2. pgk-GFP/Td donors: The constitutive expression (CE) NH-donor constructed previously [16] was used directly and named pgk-GFP_donor_ in this study. The eGFP coding sequence in pgk-GFP_donor_ was replaced with tdTomato to generate the pgk-Td_donor_.
3. 5’GFP/Td donors: The ires element was removed from the ires-GFP_donor_, using MluI at 5′ and MscI at 3′, to generate the 5′GFP_donor_. Subsequently, the eGFP coding sequence in 5′GFP_donor_ was replaced with the tdTomato gene using MfeI at 5′ and NsiI at 3′ to generate the 5′Td_donor_.

### Transfection for gene disruption and knock-in analysis

Insertional disruption of GFP transgene: LO2 cells carrying GFP transgene at *GAPDH* 3′-UTR were established as previously described [16]. Cells were seeded in 12-well plates at a density of 5 × 10^5^ cells/well and were transfected using Lipofectamine 3000 (Thermo Fisher Scientific) following the manufacturer’s instructions. A total of 2.0 μg DNA (0.8 μg Cas9 + 0.4 μg sgRNAs + 0.8 μg ires-Td_donor_) was transfected into each well. sgRNAs used included 0.2 μg sg-A and 0.2 μg eGFP-specific sgRNA. The cells were passaged 2–3 times before fluorescence imaging (Olympus IX83 Inverted Microscope) and FACS analysis (BD Aria Fusion Cell Sorter).

Gene disruption by simultaneous knock-in of dual ires-GFP/Td_donor_, pgk-GFP/Td_donor_, or 5′GFP/Td_donor_: wild-type LO2 cells were cultured as previously described [16] and were transfected with a total of 2.0 μg of DNA (0.8 μg Cas9 + 0.4 μg sgRNAs + 0.8 μg donors mixture) using Lipofectamine 3000 as described above. For each transfection, sgRNAs used included an sg-A and a site-specific sgRNA targeting different candidate gene loci (0.2 μg for each). When more than one donor was used, the amount of total plasmids was divided equally according to the number of donors. Transfected cells were passaged 5–7 times after transfection with 5′GFP/Td_donor_ and pgk-GFP/Td_donor_, 2–3 times for ires-GFP/Td_donor_, before FACS analysis (BD Aria Fusion Cell Sorter and LSRFortessa Cell Analyser).

### Genomic DNA extraction and genome PCR

Genomic DNA was extracted from cultured cells using the TIANamp Genomic DNA Kit (Tiangen) following the manufacturer’s instructions. Approximately 200–500 ng of genomic DNA was used for each PCR reaction. DreamTaq polymerase (Thermo Fisher Scientific) was used for PCR screening to detect insertional disruption of target genes. The Phusion High-Fidelity DNA polymerase kit (New England Biolabs) was used to amplify the integration junctions for sequencing. All the primers used for genome PCR are listed in Additional file 1: Table S3.

### RNA extraction, reverse transcription, and quantitative real-time PCR

Total RNA from cultured cells was isolated using TRIzol reagent (Thermo Fisher Scientific), before being reverse-transcribed into cDNA using a High Capacity cDNA Reverse Transcription Kit (Thermo Fisher Scientific). PCR was then performed using the Phusion High-Fidelity DNA polymerase kit (New England Biolabs), and quantitative real-time RT-PCR was performed using the SYBR® Premix Ex Taq kit (Takara) in 7900HT Fast Real Time PCR system (Applied Biosystems). Measurements of transcripts were normalized to *GAPDH*, and samples were run in triplicates. All primers used for RT-PCR analysis are provided in Additional file 1: Table S3. Dn-F1, Dn-F2, and Dn-R represent primers binding to donor sequence at different locations. To achieve optimal PCR amplification, we used two different forward primers binding at donors, Dn-F1 and Dn-F2, in Fig. 2a and Fig. 3a respectively. For *FAT10* RT-PCR, wild-type LO2 cells and *FAT10*-disrupted clones were treated with 20 ng/ml TNFα and 100 ng/ml INFγ for 48 h before RNA extraction.

### TA-ligation and sequencing

PCR fragments amplified from genomic DNA or cDNA were purified using the MEGAquick-spin Total Fragment DNA Purification Kit (iNtRON) and were then ligated into pGEM-T easy vectors (Promega) following the manufacturer’s instructions. Positive clones were verified by sequencing [16].

### Ectopic expression of *FAT10* and *CtIP* to rescue the *FAT10−/−* and *CtIP−/−* cells

Full-length *FAT10* cDNA and *CtIP* cDNA were cloned by RT-PCR from human LO2 cell line and inserted into pcDNA3 vector under the control of CMV promoter. The plasmids were verified by sequencing, and then transfected into obtained *FAT10−/−* and *CtIP−/−* cell clones for functional rescue.

### Western blot

Cell lysates were prepared as described previously [16]. Samples with 10 μg protein each were resolved by SDS/PAGE and subsequently transferred to polyvinylidene difluoride membranes (Bio-Rad). Membranes were blocked with 5% non-fat dry milk in PBST buffer for 1 h at room temperature and then incubated with antibodies against ULK1 (Cell Signaling, Cat# 8054, RRID: AB_11178668), FAT10 (LifeSpan BioSciences, Cat# LS-C341638, RRID Number not available), CtIP (Abcam, Cat# ab155988, RRID Number not available), GFP (Santa Cruz, Cat# sc-9996, RRID: AB_627695), or β-actin (Santa Cruz, Cat# sc-47,778, RRID: AB_2714189) overnight. Membranes were washed three times with PBST buffer and incubated with HRP-conjugated goat anti-mouse (Thermo Fisher Scientific, Cat# G-21040, RRID: AB_2536527) or goat anti-rabbit (Thermo Fisher Scientific, Cat# G-21234, RRID: AB_2536530) antibodies. Signals were detected using the Amersham ECL select western blotting detection kit (GE Health Care Life Sciences) and exposed to Super RX-N film (Fuji).

### Chromosome count

Wild-type LO2 cells were cultured as previously described [16], till 70–80% confluence. The cells were then treated with colcemid for mitotic arrest and harvested by standard hypotonic treatment and methanol/acetic acid (3:1) fixation as described previously [44]. Slides were prepared by standard air-drying method on a pre-cleaned slide. Mitotic cells at metaphase were then imaged, and the chromosome numbers in each cell were examined by experienced cytogenetic specialists.

### Fluorescence in situ hybridization (FISH)

Wild-type LO2 cells were cultured in 100 mm culture dish till 70–80% confluence. The cells were then trypsinized and swollen by 75 mM KCl treatment for 20 min at 37 °C and fixed by cold methanol/acetic acid (*v*/*v*, 3:1) for 5 min three times. Gene-specific BAC clones were purchased from CHORI, BACPAC Resources Center [45]. One-microgram DNA of each BAC clone was labeled with spectrum green dUTP or spectrum orange dUTP using the Nick Translation Kit (Abbott molecular) following the manufacturer’s instructions, to generate a gene-specific probe. Commercial probes or BAC clones located near centromere or sub-telomere region in the same chromosome of a target gene were used as reference probes (Additional file 1: Table S1). Dual-color in situ hybridization was then performed in LO2 cells for each targeted gene, combined with a reference probe located in the same chromosome labeled with different fluorescence color. Cells and probes were co-denatured at 75 °C for 5 min and hybridized overnight at 37 °C. After hybridization, slides were washed in 0.4× SSC/0.3% NP-40 at 72 °C for 2 min and 2× SSC/0.1% NP-40 at room temperature for 1 min. Slides were then mounted with ProLong Gold anti-fade reagent with DAPI. Fluorescence signal of spectrum green and orange were examined and captured using Zeiss microscope with a × 100 objective.

### Mitochondria staining

Wild-type LO2 and selected *ULK1*−/− clones (U-15 and U-16) were seeded on cover glass and treated with 10 μM Oligomycin (Sigma) and 1 μM antimycin A (Sigma) for 24 h. Cells were then washed with PBS and fixed with 4% PFA in PBS at 37 °C for 10 min. After washing with PBS, the cells were incubated with prewarmed 100 nM MitoTracker Red (Thermo Fisher Scientific) in PBS for 10 min at 37 °C for mitochondria labeling. Cells were then washed with PBS, and the mitochondria staining signals were examined and imaged using Olympus FV1000 confocal microscope.

### Cell viability analysis

The viability of cells after drug treatment was determined by standard 3-[4,5-dimethylthiazol-2-yl]-2,5 diphenyl tetrazolium bromide (MTT) assay as previously described [46]. Briefly, the cells were seeded at 5 × 10^3^ cells/per well in 96-well plates and grown for 24 h before treated with 20 ng/ml TNFα and 100 ng/ml INFγ for 48 h. In MTT assay, the cells were incubated in MTT (0.5 mg/ml) supplemented medium for 2 h, and then, the medium was aspirated off. Dimethyl sulfoxide (DMSO) was added to dissolve the insoluble formazan product, and the colored solution was quantified by measuring absorption at 570 nm using a reference wavelength of 630 nm by a spectrophotometer.

## Additional files

Additional file 1: **Figure S1.** Cytogenetic analysis of the human cell line LO2. **Figure S2.** NHEJ-based knock-in of ires-GFP reporter at coding exons allows tracing of the integration at target sites. **Figure S3.** Genome PCR of single-cell clones raised from targeting *ULK1* and *FAT10* genes. **Figure S4.** *CtIP*-disruption clones raised from targeted knock-in of ires donors. **Figure S5. ***CtIP*-disruption clones raised from simultaneous knock-in of dual pgk-GFP/Td donors at *CtIP* exon-7. **Figure S6. **Simultaneous knock-in of dual 5′GFP/Td_donor_ at *CtIP* 5′-UTR. Table S1. BAC clones and probes used for FISH analysis. Table S2. DNA sequences bound by sgRNAs. Table S3. Primers used for genome PCR and RT-PCR. (PDF 1413 kb)

Additional file 2: Individual data values for Fig. 2j; Individual data values for Fig. 2k; Individual data values for Fig. 3c; Individual data values for Fig. 4c; Individual data values for Fig. 5c. (XLSX 13 kb)

## References

[1] Porteus MH, Carroll D (2005). Gene targeting using zinc finger nucleases. Nat Biotechnol.

[2] Joung JK, Sander JD (2013). TALENs: a widely applicable technology for targeted genome editing. Nat Rev Mol Cell Biol.

[3] Cong L, Ran FA, Cox D, Lin S, Barretto R, Habib N, Hsu PD, Wu X, Jiang W, Marraffini LA (2013). Multiplex genome engineering using CRISPR/Cas systems. Science.

[4] Mali P, Yang L, Esvelt KM, Aach J, Guell M, DiCarlo JE, Norville JE, Church GM (2013). RNA-guided human genome engineering via Cas9. Science.

[5] Bhaya D, Davison M, Barrangou R (2011). CRISPR-Cas systems in bacteria and archaea: versatile small RNAs for adaptive defense and regulation. Annu Rev Genet.

[6] Barrangou R, Doudna JA (2016). Applications of CRISPR technologies in research and beyond. Nat Biotechnol.

[7] Hsu PD, Lander ES, Zhang F (2014). Development and applications of CRISPR-Cas9 for genome engineering. Cell.

[8] Lieber MR (2010). The mechanism of double-strand DNA break repair by the nonhomologous DNA end-joining pathway. Annu Rev Biochem.

[9] Heyer WD, Ehmsen KT, Liu J (2010). Regulation of homologous recombination in eukaryotes. Annu Rev Genet.

[10] Merkle FT, Neuhausser WM, Santos D, Valen E, Gagnon JA, Maas K, Sandoe J, Schier AF, Eggan K (2015). Efficient CRISPR-Cas9-mediated generation of Knockin human pluripotent stem cells lacking undesired mutations at the targeted locus. Cell Rep.

[11] Shalem O, Sanjana NE, Hartenian E, Shi X, Scott DA, Mikkelsen TS, Heckl D, Ebert BL, Root DE, Doench JG (2014). Genome-scale CRISPR-Cas9 knockout screening in human cells. Science.

[12] Wang L, Shao Y, Guan Y, Li L, Wu L, Chen F, Liu M, Chen H, Ma Y, Ma X (2015). Large genomic fragment deletion and functional gene cassette knock-in via Cas9 protein mediated genome editing in one-cell rodent embryos. Sci Rep.

[13] Cristea S, Freyvert Y, Santiago Y, Holmes MC, Urnov FD, Gregory PD, Cost GJ (2013). In vivo cleavage of transgene donors promotes nuclease-mediated targeted integration. Biotechnol Bioeng.

[14] Maresca M, Lin VG, Guo N, Yang Y (2013). Obligate ligation-gated recombination (ObLiGaRe): custom-designed nuclease-mediated targeted integration through nonhomologous end joining. Genome Res.

[15] Duroure K, De Cian A, Concordet JP, Del Bene F, Auer TO (2014). Highly efficient CRISPR/Cas9-mediated knock-in in zebrafish by homology-independent DNA repair. Genome Res.

[16] He X, Tan C, Wang F, Wang Y, Zhou R, Cui D, You W, Zhao H, Ren J, Feng B (2016). Knock-in of large reporter genes in human cells via CRISPR/Cas9-induced homology-dependent and independent DNA repair. Nucleic Acids Res.

[17] Zhou Y, Zhang H, Wei W (2016). Simultaneous generation of multi-gene knockouts in human cells. FEBS Lett.

[18] Egan DF, Shackelford DB, Mihaylova MM, Gelino S, Kohnz RA, Mair W, Vasquez DS, Joshi A, Gwinn DM, Taylor R (2011). Phosphorylation of ULK1 (hATG1) by AMP-activated protein kinase connects energy sensing to mitophagy. Science.

[19] Noda NN, Fujioka Y (2015). Atg1 family kinases in autophagy initiation. Cell Mol Life Sci.

[20] Wu W, Tian W, Hu Z, Chen G, Huang L, Li W, Zhang X, Xue P, Zhou C, Liu L (2014). ULK1 translocates to mitochondria and phosphorylates FUNDC1 to regulate mitophagy. EMBO Rep.

[21] Ren J, Wang Y, Gao Y, Mehta SB, Lee CG (2011). FAT10 mediates the effect of TNF-alpha in inducing chromosomal instability. J Cell Sci.

[22] You Z, Shi LZ, Zhu Q, Wu P, Zhang YW, Basilio A, Tonnu N, Verma IM, Berns MW, Hunter T (2009). CtIP links DNA double-strand break sensing to resection. Mol Cell.

[23] Yun MH, Hiom K (2009). CtIP-BRCA1 modulates the choice of DNA double-strand-break repair pathway throughout the cell cycle. Nature.

[24] Chen PL, Liu F, Cai S, Lin X, Li A, Chen Y, Gu B, Lee EY, Lee WH (2005). Inactivation of CtIP leads to early embryonic lethality mediated by G1 restraint and to tumorigenesis by haploid insufficiency. Mol Cell Biol.

[25] Raasi S, Schmidtke G, Groettrup M (2001). The ubiquitin-like protein FAT10 forms covalent conjugates and induces apoptosis. J Biol Chem.

[26] Jinek M, Chylinski K, Fonfara I, Hauer M, Doudna JA, Charpentier E (2012). A programmable dual-RNA-guided DNA endonuclease in adaptive bacterial immunity. Science.

[27] Mou H, Smith JL, Peng L, Yin H, Moore J, Zhang XO, Song CQ, Sheel A, Wu Q, Ozata DM (2017). CRISPR/Cas9-mediated genome editing induces exon skipping by alternative splicing or exon deletion. Genome Biol.

[28] Kapahnke M, Banning A, Tikkanen R. Random splicing of several exons caused by a single base change in the target exon of CRISPR/Cas9 mediated gene knockout. Cells. 2016;5(4):45. 10.3390/cells5040045 PMC5187529 27983621

[29] McClellan J, King MC (2010). Genetic heterogeneity in human disease. Cell.

[30] Chia NY, Chan YS, Feng B, Lu X, Orlov YL, Moreau D, Kumar P, Yang L, Jiang J, Lau MS (2010). A genome-wide RNAi screen reveals determinants of human embryonic stem cell identity. Nature.

[31] Koike-Yusa H, Li Y, Tan EP, Velasco-Herrera Mdel C, Yusa K (2014). Genome-wide recessive genetic screening in mammalian cells with a lentiviral CRISPR-guide RNA library. Nat Biotechnol.

[32] Li W, Xu H, Xiao T, Cong L, Love MI, Zhang F, Irizarry RA, Liu JS, Brown M, Liu XS (2014). MAGeCK enables robust identification of essential genes from genome-scale CRISPR/Cas9 knockout screens. Genome Biol.

[33] Gao Y, Xiong X, Wong S, Charles EJ, Lim WA, Qi LS (2016). Complex transcriptional modulation with orthogonal and inducible dCas9 regulators. Nat Methods.

[34] Liu SJ, Horlbeck MA, Cho SW, Birk HS, Malatesta M, He D, Attenello FJ, Villalta JE, Cho MY, Chen Y, et al. CRISPRi-based genome-scale identification of functional long noncoding RNA loci in human cells. Science. 2017;355(6320). 10.1126/science.aah7111 PMC5394926 27980086

[35] Human Splicing Finder. http://www.umd.be/HSF3/. Accessed 21 Jan 2018.

[36] Sartori AA, Lukas C, Coates J, Mistrik M, Fu S, Bartek J, Baer R, Lukas J, Jackson SP (2007). Human CtIP promotes DNA end resection. Nature.

[37] Sharpe JJ, Cooper TA (2017). Unexpected consequences: exon skipping caused by CRISPR-generated mutations. Genome Biol.

[38] Suzuki K, Yu C, Qu J, Li M, Yao X, Yuan T, Goebl A, Tang S, Ren R, Aizawa E (2014). Targeted gene correction minimally impacts whole-genome mutational load in human-disease-specific induced pluripotent stem cell clones. Cell Stem Cell.

[39] Veres A, Gosis BS, Ding Q, Collins R, Ragavendran A, Brand H, Erdin S, Cowan CA, Talkowski ME, Musunuru K (2014). Low incidence of off-target mutations in individual CRISPR-Cas9 and TALEN targeted human stem cell clones detected by whole-genome sequencing. Cell Stem Cell.

[40] Wang X, Wang Y, Wu X, Wang J, Qiu Z, Chang T, Huang H, Lin RJ, Yee JK (2015). Unbiased detection of off-target cleavage by CRISPR-Cas9 and TALENs using integrase-defective lentiviral vectors. Nat Biotechnol.

[41] Kim D, Bae S, Park J, Kim E, Kim S, Yu HR, Hwang J, Kim JI, Kim JS (2015). Digenome-seq: genome-wide profiling of CRISPR-Cas9 off-target effects in human cells. Nat Methods.

[42] Schaefer KA, Wu WH, Colgan DF, Tsang SH, Bassuk AG, Mahajan VB (2017). Unexpected mutations after CRISPR-Cas9 editing in vivo. Nat Methods.

[43] Hu J, Lei Y, Wong WK, Liu S, Lee KC, He X, You W, Zhou R, Guo JT, Chen X (2014). Direct activation of human and mouse Oct4 genes using engineered TALE and Cas9 transcription factors. Nucleic Acids Res.

[44] Feng B, Jiang J, Kraus P, Ng JH, Heng JC, Chan YS, Yaw LP, Zhang W, Loh YH, Han J (2009). Reprogramming of fibroblasts into induced pluripotent stem cells with orphan nuclear receptor Esrrb. Nat Cell Biol.

[45] CHORI, BACPAC Resources Center. https://bacpacresources.org. Accessed 7 Dec 2016.

[46] Ren J, Chen GG, Liu Y, Su X, Hu B, Leung BC, Wang Y, Ho RL, Yang S, Lu G, et al. Cytochrome P450 1A2 metabolizes 17beta-estradiol to suppress hepatocellular carcinoma. PLoS One. 2016;11(4):e0153863. 10.1371/journal.pone.0153863 PMC4836701 27093553

