# Supplementary material for: Homology-independent multiallelic disruption via CRISPR/Cas9-based knock-in yields distinct functional outcomes in human cells
Source: BMC Biol. 2018 Dec 28;16:151. doi: 10.1186/s12915-018-0616-2 (PMC6310992; doi:10.1186/s12915-018-0616-2)
Supplement: Supplementary file 1 — Figure S1. Cytogenetic analysis of the human cell line LO2. Figure S2. NHEJ-based knock-in of ires-GFP reporter at coding exons allows tracing of the integration at target sites. Figure S3. Genome PCR of single-cell clones raised from targeting ULK1 and FAT10 genes. Figure S4. CtIP-disruption clones raised from targeted knock-in of ires donors. Figure S5. CtIP-disruption clones raised from simultaneous knock-in of dual pgk-GFP/Td donors at CtIP exon-7. Figure S6. Simultaneous knock-in of dual 5′GFP/Tddonor at CtIP 5′-UTR. Table S1. BAC clones and probes used for FISH analysis. Table S2. DNA sequences bound by sgRNAs. Table S3. Primers used for genome PCR and RT-PCR. (PDF 1413 kb) [file 12915_2018_616_MOESM1_ESM.pdf]

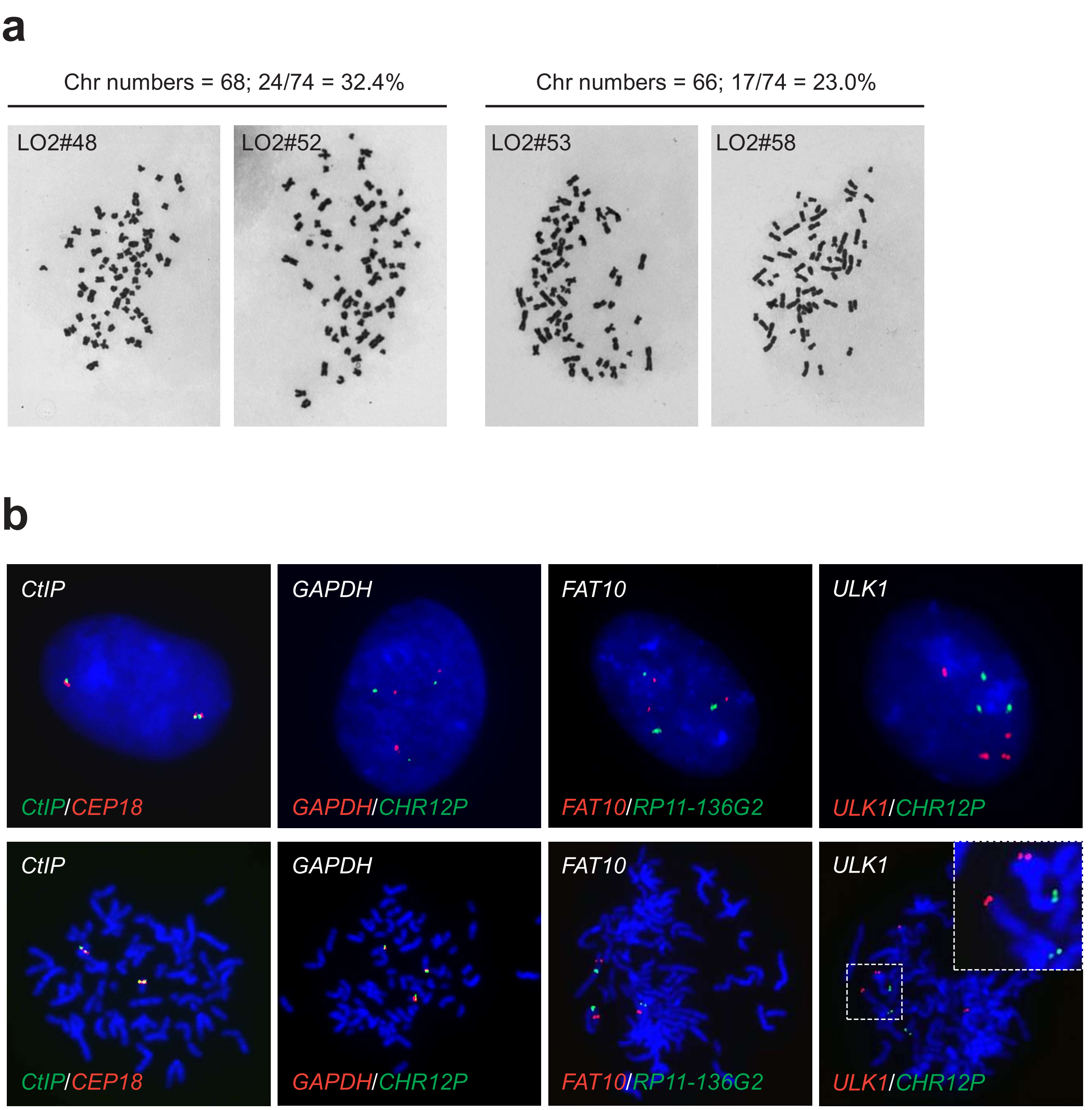

Figure S1. Cytogenetic analysis of the human cell line LO2.
a. Chromosome number analysis of the LO2 cells. Shown are selected pictures exhibiting the most commonly observed chromosome numbers. **b.** Fluorescence in situ hybridization (FISH) analysis on selected genes in the LO2 cells. Shown are results in interphase (upper row) and mitotic cells (bottom row). Probes to the target genes and reference loci are shown in their corresponding colors. BAC clones and reference FISH probes used are listed in the **Additional file 1: Table S1.**

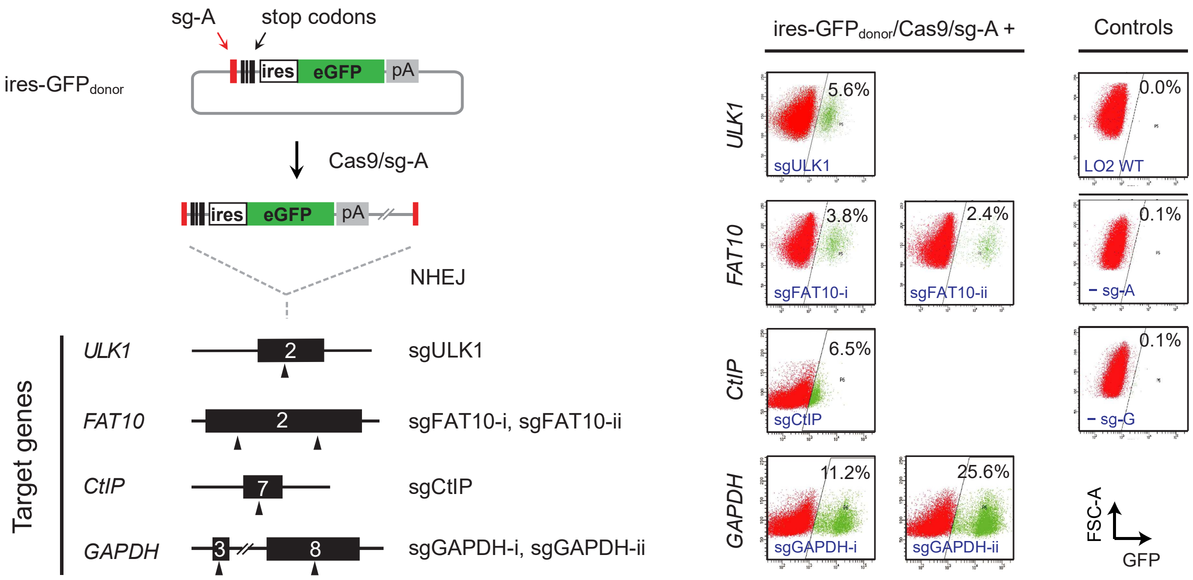

Figure S2. NHEJ-based knock-in of ires-GFP reporter at coding exons allows tracing of the integration at target sites.

NHEJ-based knock-in of ires-GFP$_{donor}$ at coding exons of *ULK1, FAT10, CtIP,* and *GAPDH* genes. Left: schematic of ires-GFP$_{donor}$ plasmid and CRISPR/Cas9-induced NHEJ-based knock-in at specific coding exons in selected target genes. sgFAT10-i and sgFAT10-ii represent two different sgRNAs targeting *FAT10* exon-2. sgGAPDH-i and sgGAPDH-ii represent two different sgRNAs targeting *GAPDH* exon-3 and exon 8 respectively. Right: FACS analysis of LO2 cells showing knock-in of ires-GFP$_{donor}$ at indicated sgRNA target sites. GFP+ cells are gated to the right in each plot. sg-G represents any sgRNA targeting *ULK1, FAT10, CtIP,* or *GAPDH* gene. Controls without sg-A or sg-G are shown in the right column.

a

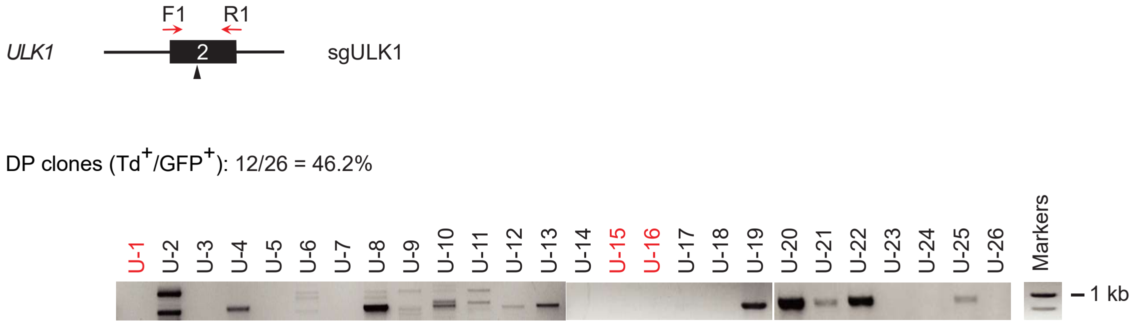

b

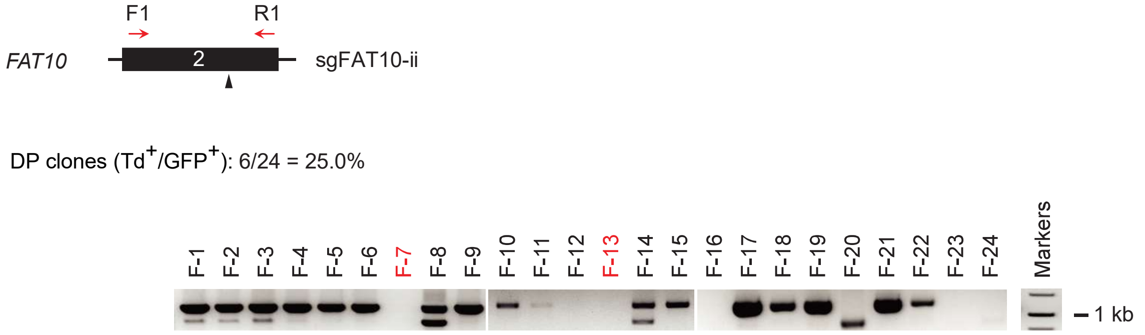

Figure S3. Genome PCR of single-cell clones raised from targeting *ULK1* and *FAT10* genes.
a. Genome PCR of single-cell clones raised from Td+/GFP+ cells produced by targeting *ULK1* exon-2. Primers used are specific to *ULK1* wild type allele, shown in **Fig. 2a**. Candidate clones selected for further analysis are indicated in red. **b.** Genome PCR of single-cell clones raised from Td+/GFP+ cells produced by targeting *FAT10* exon-2 in **Fig. 2a**. Primers shown are specific to *FAT10* wild type allele. Candidate clones selected for further analysis are indicated in red.

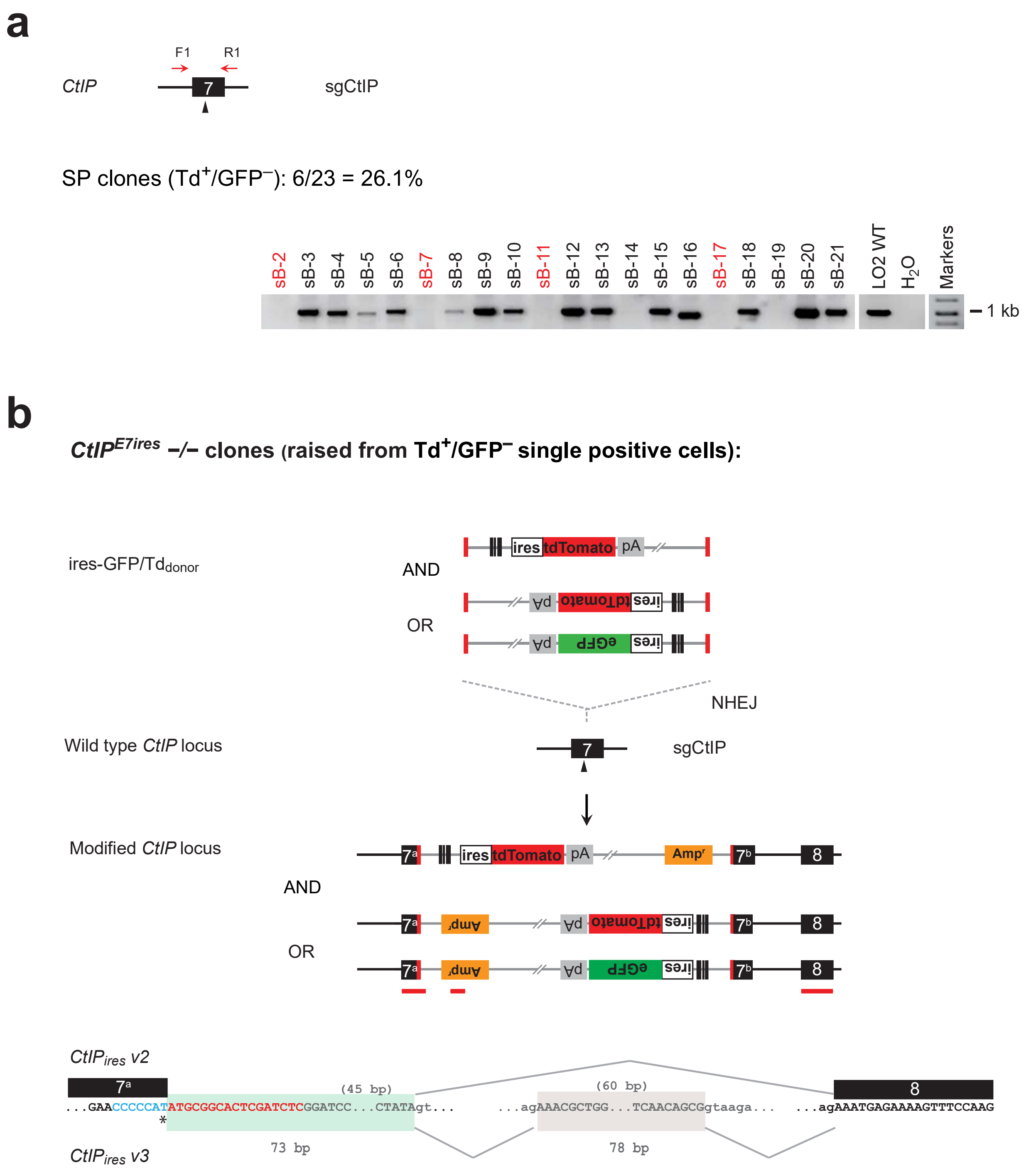

Figure S4. *CtIP*-disruption clones raised from targeted knock-in of ires-GFP/Td$_{donor}$.
a. Genome PCR analysis of single-cell clones raised from Td+/GFP− single positive (SP) cells produced by targeting *CtIP* exon-7 with ires-GFP/Td$_{donor}$. Shown are genome PCR results of single-cell clones examined. Primer binding sites at wild type *CtIP* allele are shown. Candidate clones selected for further analysis in **Fig. 3,** termed *CtIP*^{E7ires}−/− clones, are indicated in red. **b.** Schematic for reporter integrations in the *CtIP*^{E7ires}−/− clones raised from Td+/GFP− single positive (SP) cells (top panel). Red bars below the modified *CtIP* locus indicated the locations of sequences detected in the aberrant *CtIP*$_{ires}$ transcripts. Cryptic splice sites as well as splicing events involved in producing the *CtIP*$_{ires}$ transcripts are shown in bottom panel.

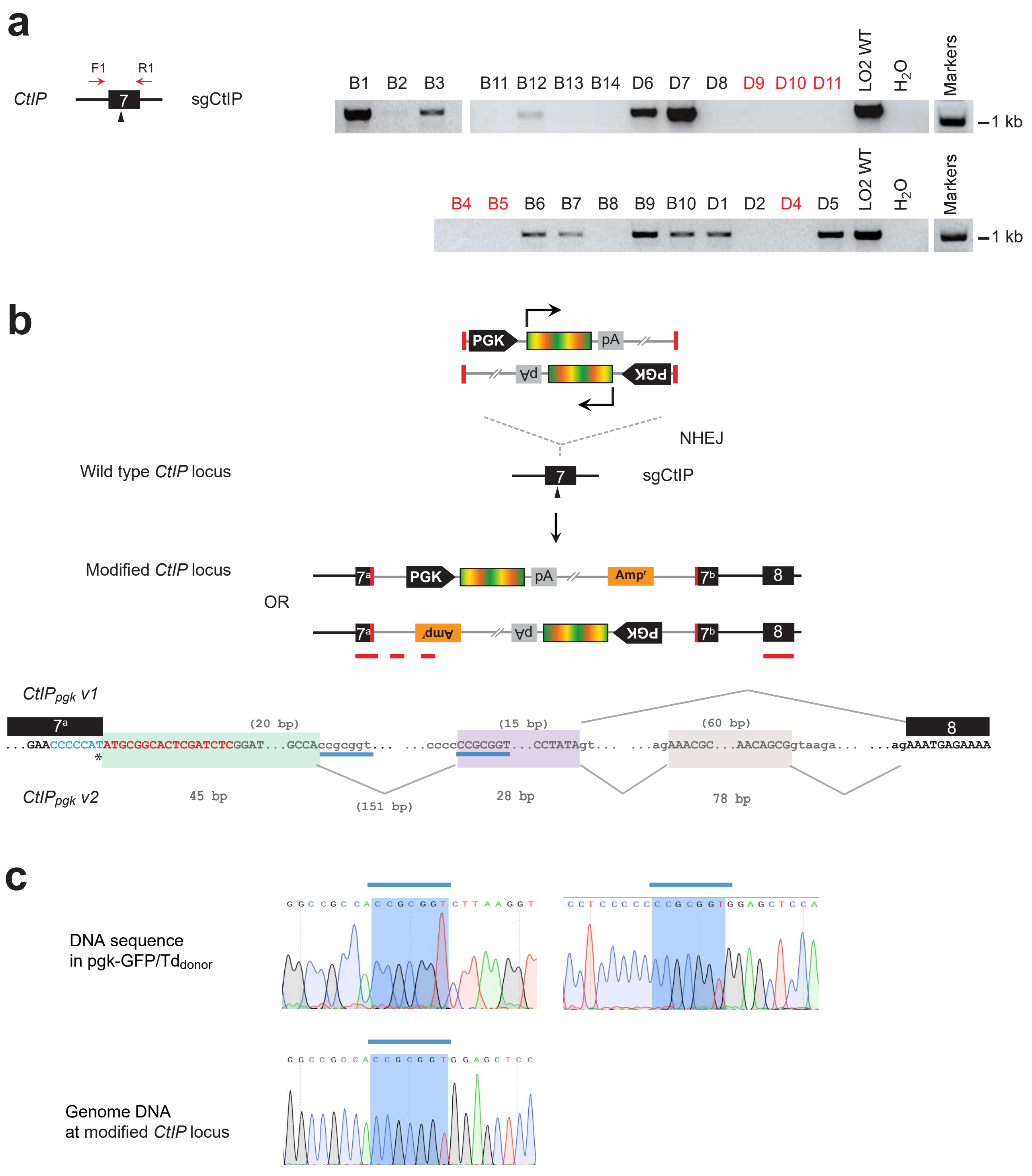

Figure S5. *CtIP*-disruption clones raised from simultaneous knock-in of dual pgk-GFP/Td$_{donor}$ at *CtIP* exon-7. **a.** Genome PCR analysis of single-cell clones raised targeted knock-in of dual pgk donors at *CtIP* exon-7. Clone B1-21 were raised from Td+/GFP+ double positive (DP) cells and clone D 1-15 were raised from Td+/GFP− single positive (SP) cells. Shown are selective results of single-cell clones examined. Primer binding sites at wild type *CtIP* allele are shown. Candidate clones selected for further analysis are indicated in red. **b.** Schematic showing the reporter integrations in *CtIP*^{E7pgk}**−/−** clones (top panel). Red bars below the modified *CtIP* allele indicated the positions of sequences detected in aberrant *CtIP*$_{pgk}$ transcripts. Cryptic splice sites as well as splicing events involved in producing the *CtIP*$_{pgk}$ transcripts are shown in bottom panel. **c.** Sequences of pgk-GFP/Td$_{donor}$ and the modified *CtIP* allele in genome carrying reversely integrated pgk-GFP/Td$_{donor}$. Shown are the microhomology sequences (highlighted in blue shade) in the donor plasmids (top panel) and in the modified *CtIP* allele in genome (bottom panel). Microhomology-based recombination occurred at genome level.

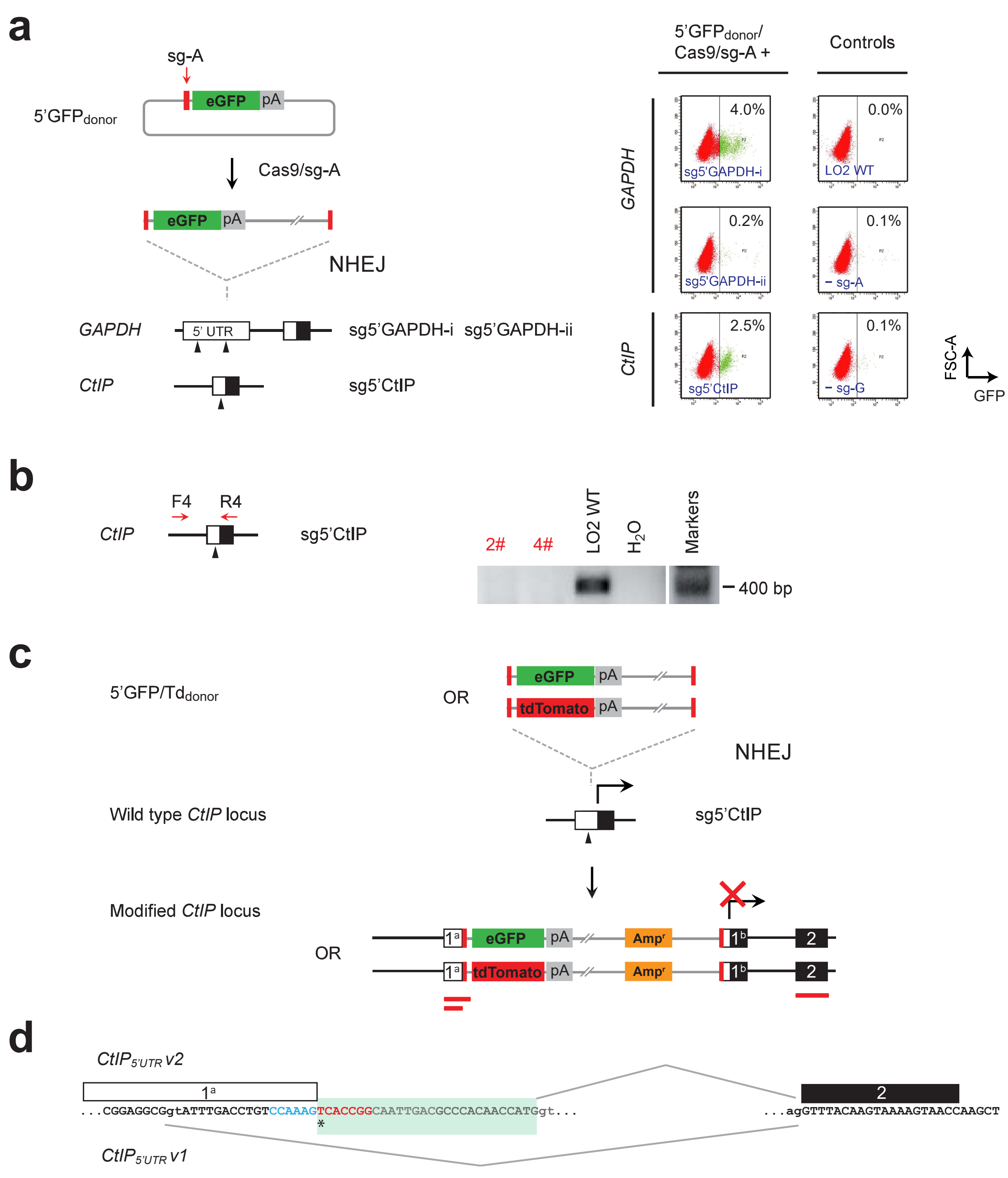

Figure S6. Simultaneous knock-in of dual 5'GFP/Td$_{donor}$ at *CtIP* 5'-UTR.
a. NHEJ-based knock-in of 5'GFP$_{donor}$ at 5'-UTR of *GAPDH* and *CtIP* genes. Left: schematic indicating the knock-in of 5'GFP$_{donor}$ at selected target sites. Right: FACS analysis in LO2 cells showing knock-in of 5'GFP/Td$_{donor}$ at the indicated target sites. GFP+ cells are gated to the right. sg-G represents any sgRNA targeting *GAPDH* or *CtIP* gene. Controls without sg-A or sg-G are shown. **b.** Genomic PCR of single-cell clones raised from Td+/GFP+ cells produced by targeting *CtIP* 5'-UTR using dual 5'GFP/Td$_{donor}$ (**Fig. 5a**). Shown are selective results. Primer binding sites at wild type *CtIP* allele are shown. Candidate clones selected for further analysis are indicated in red. **c.** Schematic showing the reporter integrations in *CtIP*$^{5'UTR}$–/– clones (top panel). Red bars below indicated the positions of sequences detected in the *CtIP*$_{5'UTR}$ transcripts.
d. Schematic showing cryptic splice sites as well as splicing events involved in producing the aberrant *CtIP*$_{5'UTR}$ transcripts. The sg-A target sequence from donor is shown in red, and sg5'CtIP target sequence in *CtIP* 5'-UTR is in blue. Other sequences from donors are indicated in grey, while other sequences from *CtIP* gene are in black. The short fragment originated from donors is highlighted with shade in light green.

Table S2. DNA sequences bound by sgRNAs.

sgRNA	Target sequences	PAM	Gene locus
sgGFP-i	CAAGCAGAAGAACGGCATCA	AGG	*GFP*
sgGFP-ii	TACCAGCAGAACACCCCCAT	CGG	*GFP*
sgULK1	GTCTCAGACGCTGCTGGGGA	AGG	*ULK1* exon-2
sgFAT10-i	GCATGTCCGTTCCGAGGAAT	GGG	*FAT10* exon-2
sgFAT10-ii	GCTTCACCACTTTCAGGGTA	AGG	*FAT10* exon-2
sgCtIP	GTTCTATGTATCGGACATGG	GGG	*CtIP* exon-7
sgGAPDH-i	GTATTGGGCGCCTGGTCACC	AGG	*GAPDH* exon-3
sgGAPDH-ii	ACTGTGGCGTGATGGCCGCG	GGG	*GAPDH* exon-8
sg5'GAPDH-i	ATTTATAGAAACCGGGGGCG	GGG	*GAPDH* 5'UTR
sg5'GAPDH-ii	GCATCTTCTTTTGCGTCGCC	AGG	*GAPDH* 5'UTR
sg5'CtIP	TAGAGGTATCAAGTCGTCTT	TGG	*CtIP* 5'UTR

Table S3. Primers used for genome PCR and RT-PCR.

Primers	Sequences
Donor	
GFP-R	AGAGATCTAGAGTTGTACTCCAGCTTGTGCCCCAGG
Td-R	CGCATGAACTCTTTGATGACC
Dn-F1	CGCCAGGGTTTTCCCAGTCACGAC
Dn-F2	CAATAGGCCGAAATCGGCAAAATCCC
Dn-R	CCTCACATTGCCAAAAGACG
ULK1	
F1	AGCCCAGATCCTCACTCCGAGAC
R1	TCAACAAACACACGGCCTAGACAG
F2	GTGGGCAAGTTCGAGTTCTC
R2	ACTCCATAACCAGGTAGACAGAATTAG
F2*	GAGAAGCACGATTTGGAGGTC
R2*	CGTACAGGGCCACGATGTTT
FAT10	
F1	AGGGAGAAGATACAATGAGGCATATCCAAC
R1	TACAATAACATGCCAGGAAGAGTAAGTTGC
F2	TTAATGACCTTTGATGCCAACCCATATGAC
R2	GAAACATAGAGTTGGGCAATATACTTCATCC
CtIP	
F1	AAATACTTTTAATTTCACCTGAAGTGC
R1	AAGCCACAAAGTGTACATATTATAACACC
F2	GAAGACGTTATTCCAGATTCACCGATAACAGC
R2	TCCAGACAGTGGTAGAGCTCATCACCAAG
F3	CAAGACACCGATTCCGCTACATTCCAC
R3	TCCTTGCCTTTTGGAGAAAATATTGCGTTG
F4	CCCCTCAATATCTGAATCCTTTCTG
R4	TGTTCATCTTGCTTAATATGCTCCACAC
F5	TTTCACAGCCTCAGAAAGTGCTCGCTTC
R5	TGTTCATCTTGCTTAATATGCTCCACACTTC
R5*	CATGTGCTTTGGCCATTGGAGATTGAC
GAPDH	
G-f1	CCAGGGCTGCTTTTAACTCTGGTAAAGTGG
G-r1	ATTTCCATTGATGACAAGCTTCCCGTTCTC

Table S1. BAC clones and probes used for FISH analysis

Chromosome ID	Target locus			Reference locus		
	Genes	BAC clone	Copy number /Probe color	Reference probe	Location	Copy number /Probe color
Chr6	*FAT10*	RP11-150A6	3 / orange	BAC: RP11-136G2	near-centromeric region	3 / green
Chr12	*GAPDH*	RP11-433J6	3 / orange	Chr12p	subtelomeric region	3 / green
Chr12	*ULK1*	RP11-394D10	4 / orange	Chr12p	subtelomeric region	3 / green
Chr18	*CtIP*	RP11-739L10	2 / green	CEP18	near-centromeric region	2 / orange
